# Loci for insulin processing and secretion provide insight into type 2 diabetes risk

**DOI:** 10.1016/j.ajhg.2023.01.002

**Published:** 2023-01-23

**Authors:** K. Alaine Broadway, Xianyong Yin, Alice Williamson, Victoria A Parsons, Emma P Wilson, Anne H Moxley, Swarooparani Vadlamudi, Arushi Varshney, Anne U Jackson, Vasudha Ahuja, Stefan R Bornstein, Laura J Corbin, Graciela E Delgado, Om P Dwivedi, Lilian Fernandes Silva, Timothy M Frayling, Harald Grallert, Stefan Gustafsson, Liisa Hakaste, Ulf Hammar, Christian Herder, Sandra Herrmann, Kurt Højlund, David A Hughes, Marcus E Kleber, Cecilia M Lindgren, Ching-Ti Liu, Jian'an Luan, Anni Malmberg, Angela P Moissl, Andrew P Morris, Nikolaos Perakakis, Annette Peters, John R Petrie, Michael Roden, Peter E. H. Schwarz, Sapna Sharma, Angela Silveira, Rona J Strawbridge, Tiinamaija Tuomi, Andrew R Wood, Peitao Wu, Björn Zethelius, Damiano Baldassarre, Johan G Eriksson, Tove Fall, Jose C Florez, Andreas Fritsche, Bruna Gigante, Anders Hamsten, Eero Kajantie, Markku Laakso, Jari Lahti, Deborah A Lawlor, Lars Lind, Winfried März, James B Meigs, Johan Sundström, Nicholas J Timpson, Robert Wagner, Mark Walker, Nicholas J Wareham, Hugh Watkins, Inês Barroso, Stephen O’Rahilly, Niels Grarup, Stephen CJ Parker, Michael Boehnke, Claudia Langenberg, Eleanor Wheeler, Karen L Mohlke

**Affiliations:** 1 Department of Genetics, University of North Carolina, Chapel Hill, NC, USA; 2 Department of Biostatistics, University of Michigan, Ann Arbor, MI, USA; 3 Center for Statistical Genetics, University of Michigan, Ann Arbor, MI, USA; 4 MRC Epidemiology Unit, Institute of Metabolic Science, University of Cambridge School of Clinical Medicine, Cambridge, UK; 5 University of Cambridge Metabolic Research Laboratories, Wellcome Trust-MRC Institute of Metabolic Science, Department of Clinical Biochemistry, University of Cambridge, Cambridge, UK; 6 Department of Computational Medicine and Bioinformatics, University of Michigan, Ann Arbor, MI, USA; 7 Institute for Molecular Medicine Finland, University of Helsinki, Helsinki, Finland; 8 Department of Internal Medicine, Metabolic and Vascular Medicine, Medical Faculty Carl Gustav Carus, Dresden, Germany; 9 Helmholtz Zentrum München, Paul Langerhans Institute Dresden (PLID), University Hospital and Faculty of Medicine, TU Dresden, Dresden, Germany; 10 German Center for Diabetes Research, Neuherberg, Germany; 11 Medical Research Council Integrative Epidemiology Unit (MRC IEU) at the University of Bristol, Bristol, UK; 12 Population Health Sciences, Bristol Medical School, University of Bristol, Bristol, UK; 13 Medical Faculty Mannheim, Heidelberg University, Mannheim, BW, Germany; 14 University of Helsinki, Helsinki, Finland; 15 Folkhälsan Research Center, Helsinki, Finland; 16 Institute of Clinical Medicine, University of Eastern Finland, Kuopio, Finland; 17 College of Medicine and Health, Exeter University, Exeter, UK; 18 Research Unit of Molecular Epidemiology, Helmholtz Zentrum München-German Research Center for Environmental Health, Neuherberg, Germany; 19 Institute of Epidemiology, Helmholtz Zentrum München-German Research Center for Environmental Health, Neuherberg, Germany; 20 Department of Medical Sciences, Clinical Epidemiology, Uppsala University, Uppsala, Sweden; 21 Department of Medical Sciences, Molecular Epidemiology and Science for Life Laboratory, Uppsala University, Uppsala, Sweden; 22 Institute for Clinical Diabetology, German Diabetes Center, Leibniz Center for Diabetes Research at Heinrich Heine University Düsseldorf, Düsseldorf, Germany; 23 Department of Endocrinology and Diabetology, Medical Faculty and University Hospital Düsseldorf, Heinrich Heine University Düsseldorf, Düsseldorf, Germany; 24 Department of Internal Medicine, Prevention and Care of Diabetes, Medical Faculty Carl Gustav Carus, Dresden, Germany; 25 Steno Diabetes Center Odense, Odense, Denmark; 26 SYNLAB MVZ Humangenetik Mannheim, Mannheim, BW, Germany; 27 Oxford Big Data Institute, Li Ka Shing Centre for Health Information and Discovery, University of Oxford, Oxford, UK; 28 Nuffield Department of Population Health, University of Oxford, Oxford, UK; 29 Wellcome Trust Centre Human Genetics, University of Oxford, Oxford, UK; 30 Broad Institute, Cambridge, MA, USA; 31 Department of Biostatistics, Boston University School of Public Health, Boston, MA, USA; 32 Department of Psychology and Logopedics, Faculty of Medicine, University of Helsinki, Helsinki, Finland; 33 Institute of Nutritional Sciences, Friedrich-Schiller-University, Jena, Germany; 34 Competence Cluster for Nutrition and Cardiovascular Health (nutriCARD), Halle-Jena-Leipzig, Germany; 35 Centre for Genetics and Genomics Versus Arthritis, Centre for Musculoskeletal Research, The University of Manchester, Manchester, UK; 36 School of Health and Wellbeing, University of Glasgow, Glasgow, UK; 37 Chair of Food Chemistry and Molecular Sensory Science, Technische Universität München, Freising, Germany; 38 Department of Medicine Solna, Division of Cardiovascular Medicine, Karolinska Institutet, Stockholm, Sweden; 39 Oxford Biomedical Research Centre, Wellcome Centre for Human Genetics, University of Oxford, Oxford, UK; 40 Institute of Health and Wellbeing, Mental Health and Wellbeing, University of Glasgow, Glasgow, UK; 41 Abdominal Center, Endocrinology, Helsinki University Hospital, Helsinki, Finland; 42 Genetics of Complex Traits, College of Medicine and Health, University of Exeter, Exeter, UK; 43 Department of Geriatrics, Uppsala University, Uppsala, Sweden; 44 Department of Medical Biotechnology and Translational Medicine, Università degli Studi di Milano, Milan, Italy; 45 Cardiovascular Prevention Area, Centro Cardiologico Monzino I.R.C.C.S., Milan, Italy; 46 Department of General Practice and Primary Health Care, Faculty of Medicine, University of Helsinki, Helsinki, Finland; 47 Folkhälsan Research Centre, Helsinki, Finland; 48 Department of Obstetrics and Gynecology, Yong Loo Lin School of Medicine, National University Singapore, Singapore, Singapore; 49 Diabetes Unit and Center for Genomic Medicine, Massachusetts General Hospital, Boston, MA, USA; 50 Programs in Metabolism and Medical & Population Genetics, Broad Institute, Cambridge, MA, USA; 51 Department of Medicine, Harvard Medical School, Boston, MA, USA; 52 Department of Internal Medicine, Diabetology, Tübingen, Germany; 53 Institute for Diabetes Research and Metabolic Diseases, Helmholtz Center Munich, University of Tübingen, Tübingen, Germany, Tübingen, Germany; 54 Population Health Unit, Finnish Institute for Health and Welfare, Helsinki, Finland; 55 PEDEGO Research Unit, MRC Oulu, Oulu University Hospital and University of Oulu, Oulu, Finland; 56 Department of Clinical and Molecular Medicine, Norwegian University of Science and Technology, Trondheim, Norway; 57 Children’s Hospital, Helsinki University Hospital and University of Helsinki, Helsinki, Finland; 58 Synlab Academy, SYNLAB Holding Deutschland GmbH, Mannheim, BW, Germany; 59 Department of Medicine, Division of General Internal Medicine, Massachusetts General Hospital, Boston, MA, USA; 60 Program in Medical and Population Genetics, Broad Institute, Cambridge, MA, USA; 61 Faculty of Medical Sciences, Newcastle University, Newcastle upon Tyne, UK; 62 Health Data Research UK, Gibbs Building, London, UK; 63 Division of Cardiovascular Medicine, Radcliffe Department of Medicine, University of Oxford, Oxford, UK; 64 Exeter Centre of Excellence for Diabetes Research (EXCEED), Genetics of Complex Traits, University of Exeter Medical School, University of Exeter, Exeter, UK; 65 MRC Metabolic Diseases Unit, Wellcome Trust-Medical Research Council Institute of Metabolic Science, University of Cambridge, Cambridge, UK; 66 Novo Nordisk Foundation Center for Basic Metabolic Research, Faculty of Health and Medical Sciences, University of Copenhagen, Copenhagen, Denmark; 67 Department of Human Genetics, University of Michigan, Ann Arbor, MI, USA; 68 Computational Medicine, Berlin Institute of Health at Charité–Universitätsmedizin Berlin, Berlin, Germany; 69 Precision Healthcare University Research Institute, Queen Mary University of London, London, UK

## Abstract

Insulin secretion is critical for glucose homeostasis, and increased levels of the precursor proinsulin relative to insulin indicate pancreatic islet beta-cell stress and insufficient insulin secretory capacity in the setting of insulin resistance. We conducted meta-analyses of genome-wide association results for fasting proinsulin from 16 European-ancestry studies in 45,861 individuals. We found 36 independent signals at 30 loci (p-value < 5x10^-8^), which validated 12 previously reported loci for proinsulin and 10 additional loci previously identified for another glycemic trait. Half of the alleles associated with higher proinsulin showed higher vs. lower effects on glucose levels, corresponding to different mechanisms. Proinsulin loci included genes that affect prohormone convertases, beta-cell dysfunction, vesicle trafficking, beta-cell transcriptional regulation, and lysosomes/autophagy processes. We colocalized 11 proinsulin signals with islet expression quantitative trait loci (eQTL) data, suggesting candidate genes including *ARSG*, *WIPI1*, *SLC7A14*, and *SIX3*. The *NKX6-3/ANK1* proinsulin signal colocalized with a T2D signal and an adipose *ANK1* eQTL signal, but not the islet *NKX6-3* eQTL. Signals were enriched for islet enhancers, and we showed a plausible islet regulatory mechanism for the lead signal in the *MADD* locus. These results show how detailed genetic studies of an intermediate phenotype can elucidate mechanisms predisposing to disease.

## Introduction

Proinsulin is a precursor to insulin that is formed in pancreatic beta cells. Some proinsulin is secreted into the plasma during insulin biosynthesis and secretion, and circulating levels of proinsulin relative to insulin are increased in individuals with type 2 diabetes (T2D) and pre-diabetes^1–3^. Elevated proinsulin relative to insulin in individuals with pre-diabetes and T2D patients may be caused by increased demand on beta cells to release insulin, thereby encouraging the premature release of granules that contain a higher ratio of proinsulin to mature insulin^3^. Conversely, reduced proinsulin-to-insulin levels could result from defects in proinsulin processing and folding prior to cleavage into insulin, early defects in vesicular processing, or altered proinsulin vs insulin degredation^4^.

Proinsulin can serve as a valuable intermediate phenotype to aid identification of genetic variations influencing hyperglycemia and T2D^5^. Additionally, the allelic effect directions on glucose vs proinsulin can help differentiate known T2D loci into those involved in beta cell stress versus defects in proinsulin processing and secretion^3,4,6–9^. Previous proinsulin genome-wide association studies (GWAS) reported 16 signals at 13 genomic loci. These studies included a meta-analysis of 10,700 discovery participants that reported 10 loci^5^, a subsequent exome array study of Finnish individuals that identified two more loci with low-frequency (minor allele frequency (MAF) < 5%) variants^10^, and a genetic study of participants with high risk for cardiovascular diseases (CVD) which identified another locus^11^. To provide a comprehensive genetic analysis of proinsulin and gain insight into glycemic trait dysregulation, we performed a large meta-analysis of proinsulin GWAS. This study quadrupled the sample size of the largest previous meta-analysis and doubled the number of proinsulin association signals, implicating candidate genes that regulate insulin processing and glucose regulation.

## Methods

### Cohort/study description

As part of the Meta-Analysis of Glycemic and Insulin traits Consortium (MAGIC), we conducted a meta-analysis of GWAS results for fasting proinsulin levels from 16 European-ancestry cohorts in up to 45,861 individuals (Table S1). Each of 16 cohorts collected trait and genotype data, assessed quality, and performed association analyses (Table S1). Each cohort performed imputation and reported all variants to Genome Reference Consortium Human Build 37/hg19^12^. Study participants who had diabetes, were on a diabetes treatment, or had fasting glucose ≥7 mmol/L, 2-hour glucose ≥11.1 mmol/L, or hemoglobin A1c (HbA1c) ≥6.5% (48 mmol/mol) were excluded. Fasting proinsulin values (pmol/L) were natural logarithm transformed and analyses adjusted for age, sex, population structure, and natural logarithm of fasting insulin (study-level details of fasting requirements, sample collection, and population structure adjustments are in Table S1). Study analysts ran models adjusted and unadjusted for body mass index (BMI). To control for type I error rate of low-frequency variants and to fully remove trait-covariate correlations, covariate adjustment was performed in two steps^13^. Analysts first modeled natural logarithm of fasting proinsulin on all covariates, then inverse normal transformed the residuals. Analysts then modeled the inverse normally transformed residuals on the covariates again and used these residuals in the final regression analysis. Analysts used an additive-model in a linear/linear mixed-model framework using software including EPACTS, rvtests, and PLINK^14–16^.

### Study-level quality control (QC)

Central analysts assessed each cohort input file for quality control using EasyQC^17^. We excluded variants with low minor allele count (<3) or low minor allele frequency (MAF < 0.005), low call rate (<95%), deviation from Hardy-Weinberg equilibrium (HWE) (p-value<0.00001), low imputation quality (r^2^ <0.3), or exceptionally large effect standard errors (standard error >10). We also examined quantile-quantile (QQ) plots by frequency bins, assessed trends in standard errors relative to sample size, and checked allele frequencies relative to their frequency in HRC. Systematic QC issues for a study were resolved prior to inclusion in the meta-analyses.

### GWAS meta-analysis

We performed a fixed-effects inverse-variance weighted meta-analysis using METAL^18^ using effect size estimates and standard error. We applied genomic-control (GC) on summary statistics for each study and also following the meta-analysis. Post-meta-analysis inclusion criteria required that variants were represented by at least one quarter of the maximum sample size, in at least two studies, and had an overall MAF > 0.005; we analyzed 9,533,557 variants. We defined a locus as a lead variant p-value <5x10^-8^ and all variants within 500 kb. We used SWISS (https://github.com/statgen/swiss) to identify the lead variant for each locus and combined adjacent loci whose lead variants exhibited linkage disequilibrium (LD) (r^2^ > 0.4) to form an extended locus region. All linkage disequilibrium (LD) calculations are based on 1000 Genomes Europeans unless otherwise noted. We estimated the proportion of variance explained by each variant as 2β^2^f(1-f) where β is the effect size from METAL and f is the average effect allele frequency in the meta-analysis. We summed the variants’ proportion of variance to estimate total fasting proinsulin variance explained.

### Approximate conditional analysis

To identify conditionally distinct signals within a locus, we performed approximate conditional analysis using GCTA^19,20^. To reduce collinearity, we excluded any variant from designation as part of a distinct signal if its multiple regression r^2^ on the other selected variants was greater than 0.8. Since no lead proinsulin variant was within 1 Mb of another, and we noted regions of extended LD surrounding at least one lead proinsulin variant, we analyzed all variants within 1 Mb of each lead variant or the extended locus region, whichever was larger. Given that GCTA depends on use of a large representative LD reference panel, we compared results from three genotype-level reference panels: METSIM (n=10,070)^21^ and Fenland (n= 8,925)^22^ are the two largest studies in the meta-analysis that combined represent 38% of the total sample size, and Electronic MEdical Records and GEnomics (eMERGE, dbGaP Study Accession: phs000888.v1.p1) (n=6,795) is a European-only general research subset^23^. We defined a signal as conditionally distinct if a variant from GCTA representing the signal was identified with at least two of the three reference panels and the variants were proxies of each other (r^2^ > 0.8). We additionally required variants to have consistent MAF across the summary data and the reference panels; the MAF of rs181143493 near *ARAP1* was 0.12 in the proinsulin summary results and <0.01 in both the METSIM and eMERGE reference panels and therefore was excluded. Due to limitations in approximate conditional analysis with an external LD reference panel, we report at most three signals within a locus.

### Colocalization with glycemic traits

We assessed signal overlap, or colocalization, between the 36 primary and secondary proinsulin signals and the conditionally distinct signals reported by three T2D studies: the European-ancestry component of DIAbetes Meta-ANalysis of Trans-Ethnic association studies (DIAMANTE EUR)^24^, the full multi-ancestry DIAMANTE analysis (DIAMANTE TA)^25^, Asian Genetic Epidemiology Network (AGEN)/East Asian ancestry (EAS) DIAMANTE^26^, and four European-ancestry Meta-Analysis of Glucose and Insulin-related traits Consortium (MAGIC) glycemic traits: fasting glucose, fasting insulin, HbA1c, and glucose 2 hours after a glucose challenge^27^. We tested for colocalization using two strategies: colocalization based on pairwise LD (*r^2^* > 0.8) between the lead proinsulin variant and the lead variant for another trait, and a Bayesian multi-trait colocalization approach, either HyPrColoc^28^ or coloc^29^. Due to differences in ancestry across proinsulin versus AGEN and DIAMANTE TA, we ran HyPrColoc with proinsulin, DIAMANTE EUR, and the four MAGIC traits. We observed some issues with sensitivity using HyPrColoc, including unstable trait clusters and deflated PPFC values when multiple signals in the cluster are marginally significant. While multi-trait HyPrColoc provided a beneficial first-pass assessment for colocalization, sensitivity analyses using pairwise colocalization helped fine-tune the specific studies that colocalized with our proinsulin data. Therefore, we compared HyPrColoc’s multi-trait performance against a series of 2-trait colocalization analyses (i.e., proinsulin and results for only one of the other 5 traits).

We performed HyPrColoc analyses using predefined, approximately independent LD blocks, and included all traits that had at least one variant with a p-value <10^-4^ within the LD block^30^. We selected the default HyPrColoc settings (prior.1 = .0001, prior.2 = 0.98). We then ran sensitivity analyses, varying the regional alignment thresholds from 0.6 to 0.9, the alignment thresholds from 0.6 to 0.9, and the prior.2 from 0.98 to 0.995. Since Bayesian colocalization methods may be sensitive to differences in ancestry across studies, we separately performed 2-trait coloc analyses between proinsulin signals and genome-wide significant DIAMANTE TA signals then proinsulin and AGEN T2D signals. We selected coloc’s default prior probability of colocalization of 1x10^-5^ and ran sensitivity analyses varying the priors across 100 values. The cumulative sensitivity score for HyprColoc and coloc was the proportion of scores that identified a colocalization and ranged from 0 (no sensitivity tests identify colocalization) to 1 (all sensitivity tests identify colocalization). Given limitations in colocalization approaches, we considered both Bayesian methods and LD; we considered the signals colocalized if the Bayesian posterior probability of colocalization was >0.6 and either the sensitivity score was >0.4 or LD *r^2^* > 0.8 between lead variants.

### Characterization of proinsulin locus effect directions to other glycemic traits

To assess the direction of effect of proinsulin signals on T2D and common glycemic traits, we looked up associations for proinsulin lead variants in the summary results for T2D in aforementioned three studies and the four glycemic traits in MAGIC studies^24–27^. If a proinsulin lead signal was associated with T2D or fasting glucose (p-value <10^-4^) or at least two outcomes in the same direction at a more lenient p-value threshold (p-value <0.01), we reported the consensus direction of effect. To evaluate proinsulin variant association with additional glycemic traits, we performed similar look ups in the summary results for 34 glycemic traits analyzed in the METSIM study (Table S2)^10^; briefly, these traits included proinsulin, glucose, and insulin levels at fasting, after an oral glucose tolerance test (30 minutes to 120 minutes), and calculated areas under the curve measures as well as C-peptide, HbA1c, insulinogenic index, Matsuda index, and T2D. We analyzed the 34 traits as a subset of a total of 1076 baseline traits for association with variants imputed using a reference panel from a subset of METSIM with whole genome sequencing^31^. For glucose and insulin metabolic traits, we excluded individuals known to be diabetic at baseline. For each quantitative trait, we inverse normalized the trait, regressed on covariates (see Table S2 for covariates per trait), and inverse normalized the residuals. We carried out single-variant association tests using a linear mixed model in SAIGE v0.39 (https://github.com/weizhouUMICH/SAIGE) on the normalized residual trait values.

We additionally looked up proinsulin lead variants for loci not identified in T2D or glycemic trait association results. We used genetics.opentargets.org to find significant associations (p-value < 5x10^-4^) with the lead variants at these loci^32,33^. The online resource identifies associations from the GWAS Catalog^34^, Neale lab UK Biobank summary statistics (http://www.nealelab.is/uk-biobank/), SAIGE UK Biobank summary statistics^35^, and FinnGen Summary statistics^36^.

### Candidate genes

We obtained nearby gene’s islet expression specificity index (iESI) deciles^37^. iESI deciles indicate the extent to which genes are both highly expressed in islets as well as the specificity for islet expression versus ubiquitous expression across other tissues, with values near zero representing genes that have low islet specificity or low expression in islets and values near 10 representing genes whose expression is highly specific to islets. We define high iESI genes as those with a decile above 7. We consolidated gene labels across sources using Entrez gene symbols.

Next, we performed colocalization of proinsulin signals with two eQTL datasets. First, a human islet RNA-seq-based eQTL study from the InsPIRE consortium (n=420)^38^, which reported significant eQTL for 4,312 genes (FDR <1%), and second, a subcutaneous adipose tissue RNA-seq study from 434 Finnish men in the METSIM study^39^, which reported at least one significant eQTL at 9,687 genes (FDR <1%). We used LD and HyPrColoc to test for colocalizations with genes within 1 Mb of each lead proinsulin variant; as described in the previous section, we used a multi-study framework with proinsulin, European-ancestry DIAMANTE ^24^, MAGIC glycemic traits^27^, and one eQTL gene at a time, as well as testing with only proinsulin and each gene. We considered the signals colocalized if HyPrColoc posterior probability for colocalization (PPFC) scores were >0.6 and either the sensitivity score was >0.4 or LD *r^2^* > 0.8. We plotted signals using LocusZoom^40^. Additionally, we performed summary Mendelian randomization (SMR)^41^ to begin assessing potential causal relationships by using the genetic variants as an instrumental variable to test for the causative effect of gene expression on proinsulin. To account for multiple hypothesis testing, we used a Bonferroni-corrected significance threshold. To evaluate evidence of pleiotropy from linkage between two distinct causal variants, we ran heterogeneity in dependent instruments (HEIDI) as part of the SMR analysis.

### Identification of extended credible set variants

We determined 99% credible sets using regions ± 500 kb around each lead variant, using the following equation for Bayes factors:

$$ln(BF)\propto 0.5\frac{\beta^{2}}{SE^{2}},$$

where *β* and SE are the effect sizes and standard errors from the meta-analysis ^42^. For loci with multiple significant signals, we used the approximate conditional analysis option in GCTA, using eMERGE as the reference panel, to define credible sets. Variants with a low posterior probability are less likely to be causal; however, variants that are not represented or poorly represented in the meta-analysis may erroneously be excluded from consideration as a putative causal variant. We therefore extended the credible set to include all variants in high LD (r^2^ > 0.8 in 1000 Genomes European) with the lead variant. This approach recognizes variants that are not included in the meta-analysis due to analytic or technical factors (e.g. indels are not imputed by HRC and variants with MAF < 0.5%), as well as variant that are poorly represented in our meta-analysis due to factors such as low sample size.

### Coding and regulatory elements

To identify potential candidate genes for each signal, we considered protein-coding genes within ~100 kb of the signal’s lead variant^43^, with special attention to genes for which a coding variant is included in a signal’s extended credible set and those that are highly and specifically expressed in islets. To identify genes through coding effects, we obtained annotation for all variants in our extended credible set using Variant Effect Predictor (VEP)^44^, Sorting Intolerant from Tolerant (SIFT)^45^, PolyPhen-2^46^, Combined Annotation Dependent Depletion (CADD)^47,48^, and MutationAssessor^49^. For all functional predication tools, we selected default thresholds.

We tested proinsulin signals for regulatory element enrichment using the following epigenomic annotations: chromatin states in islets, adipose, and skeletal muscle^50^, bulk ATAC-seq peaks^38,51^, islet sn-ATAC cluster peaks^52^, and other islet chromatin annotations^53^. We used the Genomic Regulatory Elements and GWAS Overlap algorithm (GREGOR) to evaluate global enrichment of proinsulin-associated variants in epigenomic regulatory features^54^. GREGOR observes the signal overlap in annotated regulatory data among lead GWAS variants or their LD proxies (r^2^ > 0.8) relative to expected overlap-based control variants matched to index variants for number of variants in LD, minor allele frequency, and distance to nearest gene.

### Transcriptional activity assays

#### Cell culture

We cultured INS1-derived rat insulinoma pancreatic beta-islet 832/13 cells (provided by C. Newgard, Duke University, Durham, NC) in RPMI 1640 medium (Corning, NY) supplemented with 10% FBS, 10 mM HEPES, 2 mM L-glutamine, 1 mM sodium pyruvate, and 50 μM 2-mercaptoethanol, and we cultured murine insulinoma MIN6 cells (provided by C. Rhodes, Joslin Diabetes Center, Boston, MA) in high-glucose DMEM (Sigma-Aldrich, St. Louis, MO) supplemented with 10% FBS, 1 mM sodium pyruvate, and 100 μM 2-mercaptoethanol. All cells were maintained in a humidified incubator at 37°C with 5% CO2, and prior to transfection, both cell lines tested negative for *Mycoplasma* contamination in accordance with the MycoAlert *Mycoplasma* Detection Kit (Lonza, Morristown, NJ).

#### Transcriptional reporter assays

To test for allelic differences in transcriptional activity, we performed dual-luciferase reporter assays as previously described^55^. We used genomic DNA of individuals homozygous for the reference or alternate alleles to amplify fragments surrounding rs10501320, cloned amplicons into the firefly luciferase reporter vector pgL4.23 (Promega, Madison, WI), and sequence-confirmed five purified clones for each allele, in each orientation (Azenta, Research Triangle Park, NC); alleles at additional variants within each amplicon were kept consistent (Table S3). Twenty-four hours prior to transfection, we seeded 832/13 and MIN6 cells in 24-well plates (200,000 cells per well). Upon reaching 90% confluence, we transfected 832/13 cells in duplicate with 500 ng of plasmid DNA and 1 μL of Lipofectamine 3000 (Thermo Fisher Scientific, Waltham, MA) per well, and we transfected MIN6 cells in duplicate with 250 ng of plasmid DNA and 1 μL Lipofectamine LTX (Thermo Fisher Scientific) per well; we co-transfected both 832/13 and MIN6 cells with 80 ng of phRL-TK *Renilla* (Promega) per well. We used two independent preparations of empty vector pgL4.23 as negative controls. After 48 hours, we performed dual-luciferase reporter assays (Promega), normalized luciferase to *Renilla*, and calculated fold-change relative to empty vector controls using two-sided t-tests assuming equal variance (α=0.05). We independently repeated transfections on different days and observed consistent results. Results show ten biological replicates (separate transfections) and two averaged technical replicates (luciferase and *Renilla* readings).

## Results

### Identification of proinsulin association signals

We identified 28 loci associated at genome-wide significance (p-value < 5x10^-8^) with proinsulin adjusted for BMI, including 16 loci >500 kb away from a previously-reported proinsulin association (Table 1, Table S4, Figures S1-S2). Combined, the 28 lead variants explained an estimated 8.9% of the total proinsulin variance in the meta-analysis, with the estimated percent of trait variance explained by each variant ranging from 2.1% (*STARD10*) to 0.07% (*JARID2*).

Association results for fasting proinsulin without BMI adjustment yielded results similar to those obtained in the BMI-adjusted analysis (Pearson correlation of effect estimates = 0.97; Figure S3, Table S5). Variants at two additional loci, *SLC2A10* and *BCL11A*, which narrowly missed the significance threshold in the analysis with BMI adjustment (p-value = 6x10^-8^ and 1.5x10^-7^, respectively) attained genome-wide significance in the analysis without BMI adjustment (Table 1).

We performed subsequent approximate conditional analysis and identified six additional signals at genome-wide significance located within 500 kb of the lead variant of five known proinsulin loci near *STARD10*, *MADD*, *PCSK1*, *SGSM2*, and *DDX31* (Table 2, Table S6, Figures S4-S5).

We identified three previously-reported signals near *MADD*, including one signal that consists of a proinsulin-associated^10^ nonsense variant (rs35233100) that is now genome-wide significant after conditioning on the lead signal (rs10501320). Both the primary and secondary signals at the *SGSM2* locus have been previously reported^5,10,11^. We also identify secondary signals located near *STARD10*, *PCSK1*, and *DDX31*. At *DDX31*, although both signals (rs368476 and rs7864386) were within 50 kb of the previously-reported female-specific *DDX31* signal (rs306549)^11^, neither was in high LD with the previously-reported lead variant (r^2^< 0.1, Figure S5)^5^, validating the *DDX31* locus, but not the previously-reported signal. For subsequent analyses, unless otherwise stated, we included the 28 primary signals and six conditionally distinct signals for proinsulin adjusted for BMI, as well as the two signals for proinsulin not adjusted for BMI, for a total of 36 signals at 30 loci.

This meta-analysis replicated four low-frequency (MAF < 0.05) proinsulin-associated signals originally identified in an exome array analysis of Finnish participants in the METSIM exome study^10^ (Table S7, Figures S6-S7). We validated missense or nonsense lead variants in *TBC1D30*, *SGSM2*, and *MADD*; all of which were genome-wide significant in the meta-analysis even after excluding METSIM. The signal at the *KANK1* locus was only genome-wide significant in the full meta-analysis (lead variant rs146375546, p-value = 4.3x10^-11^), as the lead variant is rare in general European-ancestry populations but enriched in Finnish ancestry populations (1000G MAF = 0.003 in 1000G European ancestry populations vs 0.015 in the Finnish population). The replications of associations at the four low-frequency variants highlight the utility of exome arrays in finding low-frequency variants and the challenges in replicating variants that are not equally represented across populations.

### Proinsulin signals and other glycemic traits

We compared all 36 proinsulin signals described above to up to 568 GWAS signals identified for T2D^24–26^ and up to 218 signals in four glycemic traits including fasting and 2-hour glucose, HbA1c, and fasting insulin^27^ (Tables S8-S10). We performed colocalization analysis and identified colocalizations for 15 proinsulin signals with signals for T2D (N=12) or glycemic traits (N=9): 6 previously-known proinsulin signals near *STARD10*, *MADD*, *TCF7L2*, *SGSM2*, *SLC30A8*, and *C2CD4A/B*, and 9 additional proinsulin signals near *SIX3*, *TLE1*, *RNF6*, *PAM*, *NKX6-3*, *FAM185A*, *BCL11A*, *GIPR*, and *FAM46C*. We also identified colocalizations between an additional 10 T2D or glycemic trait loci that were associated with proinsulin at a less stringent significance threshold (5x10^-8^ < p-value < 1x10^-4^) (Table S8). Eight proinsulin loci (*STX16*, *DLC1*, *SLC7A14*, *WIPI1*, *JARID2*, *SLC2A10*, *ELAPOR1*, and *PCSK2*) were not colocalized with T2D or any glycemic trait.

**Figure 1:**
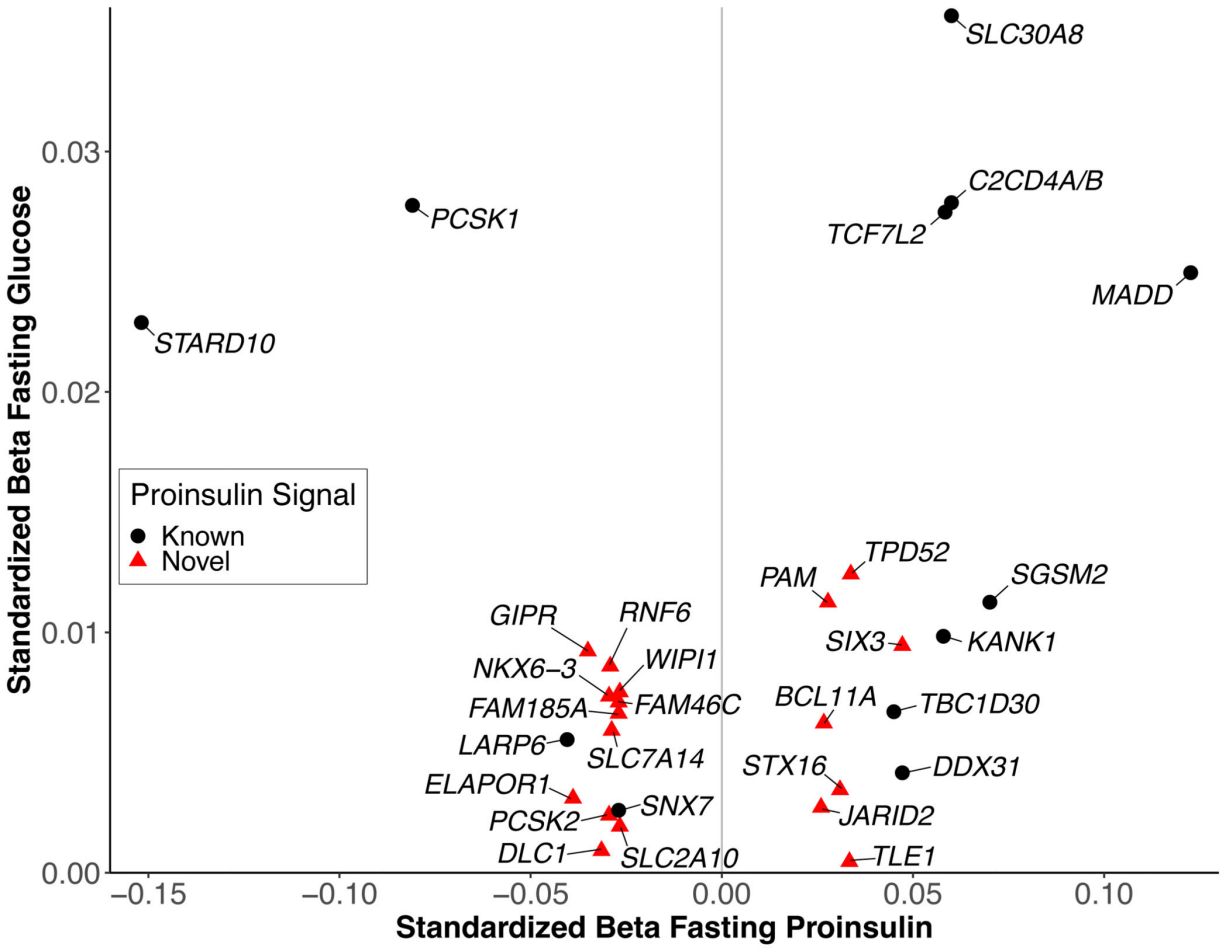
Direction of allelic effect of fasting glucose vs. fasting proinsulin. Standardized effect sizes for lead variants are shown from this study compared to fasting glucose from Chen (2021)^27^. Left of the vertical line, alleles associated with higher fasting glucose and lower proinsulin; right of the vertical line, alleles associated with higher fasting glucose and higher proinsulin.

We obtained the direction of allelic effect of the 30 lead proinsulin leads on fasting glucose^27^ and more than 30 other related glycemic traits including proinsulin levels after an oral glucose challenge^10^ (Figure 1, Tables S2, S10). The allele associated with higher glucose was associated with higher proinsulin for half the lead variants (15 of 30) and associated with lower proinsulin for the other half.

### Putative candidate genes

To identify potential candidate genes for each signal, we identified nearby genes, obtained their iESI deciles, and performed colocalization and SMR analyses with eQTL data (Tables S11-S14)^38,39^. Genes with high expression levels in islets, particularly those that are not highly expressed in other tissues, represent strong candidate genes for influencing the proinsulin to insulin processing pathway. These genes that are highly and specifically expressed in islets will have a high iESI values (defined as iESI decile > 7)^37^. Most (29/36) proinsulin signals fell within 100 kb of at least one gene with a high iESI (Table S11). Top iESI genes included well-documented beta-cell genes such as *MADD*, *PCSK1* and *PCSK2*^56–58^, as well as genes at loci not previously described in glycemic trait studies: *ELAPOR1* and *SLC7A14*.

To identify additional candidate genes underlying the proinsulin association signals, we colocalized them with eQTL signals^38,39^ (Table S12-13).Through colocalization with eQTL in pancreatic islets from the InsPIRE consortium^38^, we identified 11 proinsulin signals that colocalized with eQTL signals for 17 genes (Table S12); six proinsulin signals colocalized with eQTL for more than one gene. The alleles associated with higher proinsulin were associated with higher expression of eight genes (*MADD*, *RNF6*, *CDK8*, *SLC2A10*, *SNX7*, *ARAP1*, *STARD10*, and *TCF7L2*), and lower expression of nine protein coding genes or noncoding transcripts (*SIX3, SIX2, RP11-89K21.1, AC012354.6, ARSG, WIPI1, SLC7A14, FAM46C*, and *LARP6*). All 17 colocalizations also passed the experiment-wide significance threshold for SMR (p-value < 0.0029). Using HEIDI, we detected heterogeneity for just 1 gene at p-value < 0.0029: *STARD10*. While this may indicate the correlation is due to linkage rather than pleiotropy, the result may also be due to the complicated structure of this locus, which may violate the assumption of only one causal variant in the eQTL region.

Signal colocalization at the *NKX6-3/ANK1* locus provided additional data with which to interpret this complex locus. The locus includes two T2D signals^24,26^: one colocalized with the *NKX6-3* eQTL in islets^24^ and the other colocalized with an *ANK1* eQTL in adipose and muscle^26,59^.

**Figure 2:**
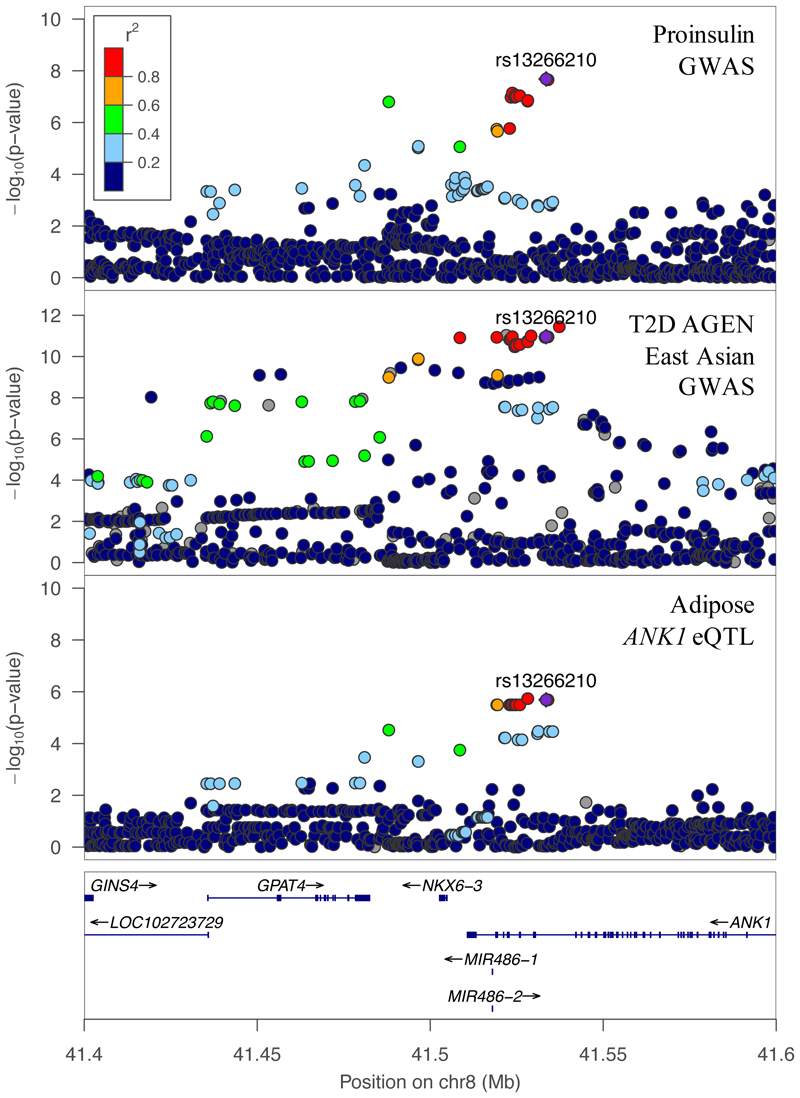
The *ANK1/NKX6-3* locus associations with proinsulin, T2D, and adipose *ANK1* expression. The proinsulin signal at this locus colocalizes with the second AGEN T2D signal and the METSIM adipose *ANK1* eQTL signal (HyPrColoc PPFC = 0.92). We used approximate conditional analysis results for the AGEN second signal in HyPrColoc as well as for the plot shown above. AGEN results colored by ASN 1000G LD reference.

*NKX6-3* is highly and specifically expressed in islets (iESI decile = 10), while *ANK1* is not (iESI decile = 2). The T2D risk alleles for the two signals were associated with lower islet *NKX6-3* expression and higher *ANK1* expression in adipose and muscle, suggesting that the signals affect T2D risk in different tissues. We observed only one proinsulin association signal at this locus. While we might have expected it to align with the proposed islet *NKX6-3* eQTL signal, it instead colocalized with the adipose *ANK1* eQTL signal (Figure 2, Figure S8, Table S13). The proinsulin lead variant rs13266210 is in strong LD with the *ANK1* eQTL (rs3802315, r^2^ = 0.84) and the East Asian AGEN T2D lead variant (rs62508166, r^2^ = 0.92), and HyPrColoc shows strong evidence of colocalization across all three studies (PPFC = 0.92). The A allele of rs13266210 is associated with increased T2D risk, higher *ANK1* expression in adipose, and lower proinsulin. At this proinsulin signal, proxy variant rs6989203 (LD r^2^ = 0.84 with rs13266210) overlaps with an islet beta-cell single nucleus ATAC peak^52^ and is in high LD with the *ANK1* eQTL site (r^2^ = 0.93). Of the two T2D signals at the *ANK1/NKX6-3* locus previously proposed to act in different tissues on different genes, the proinsulin signal colocalizes with the adipose *ANK1* signal, versus the expected colocalization with islet *NKX6-3*.

### Credible sets and variant annotation and function

We built a credible set of putative causal variants for each of the 36 signals. These 36 sets together contained 814 variants (Table S15). We extended the credible sets to include 276 additional variants exhibiting LD r^2^ ≥ 0.8 (1000 Genome European-ancestry reference) with the lead variants, including 142 variants that were unavailable in the meta-analysis and therefore could not have been included in the Bayesian credible set. Three signals had one variant in the extended credible set (*SGSM2*, *ELAPOR1*, and the second signal in *DDX31*) and 14 signals (39%) had ten variants or fewer.

The extended credible sets for 17 proinsulin signals contained coding variants (Table S16). Across all credible sets, we observed 1 nonsense, 18 missense, and 31 synonymous variants. The credible sets for 13 proinsulin signals contained at least one missense variant: seven signals in previously-identified proinsulin loci (*TBC1D30*, *PCSK1*, *KANK1*, *FAM185A*, the first and second signals at *SGSM2*, and the third signal in *MADD*), four in loci known in other glycemic trait GWAS (*SLC30A8*, *GIPR*, *FAM46C*, and *PAM*), and two that are not known proinsulin or glycemic trait genes (*ELAPOR1* and *WIPI1*).The lead variant rs74920406 at the *ELAPOR1* locus, a missense variant of low-frequency (p.His55Tyr, MAF = 0.04), was not previously associated with proinsulin or other glycemic traits but was associated with LDL (Table S17)^60^. This variant is conserved across species^48,61,62^ and has a probably damaging effect on the protein^46^. *ELAPOR1* encodes endosome-lysosome associated apoptosis and autophagy regulator 1 and inhibits beta-cell insulin signaling by accelerating recycling of the insulin receptor and insulin-like growth factor receptors^63^. The credible set for *WIPI1* contained a coding missense variant (p.Thr31Ile; rs883541). *WIPI1* is a phosphatidylinositol-2-phosphate effector gene, which encodes a component of the autophagy machinery; skeletal muscle from severely insulin resistant patients with T2D displayed decreased expression of autophagy-related genes, including *WIPI1*^64^.

**Figure 3:**
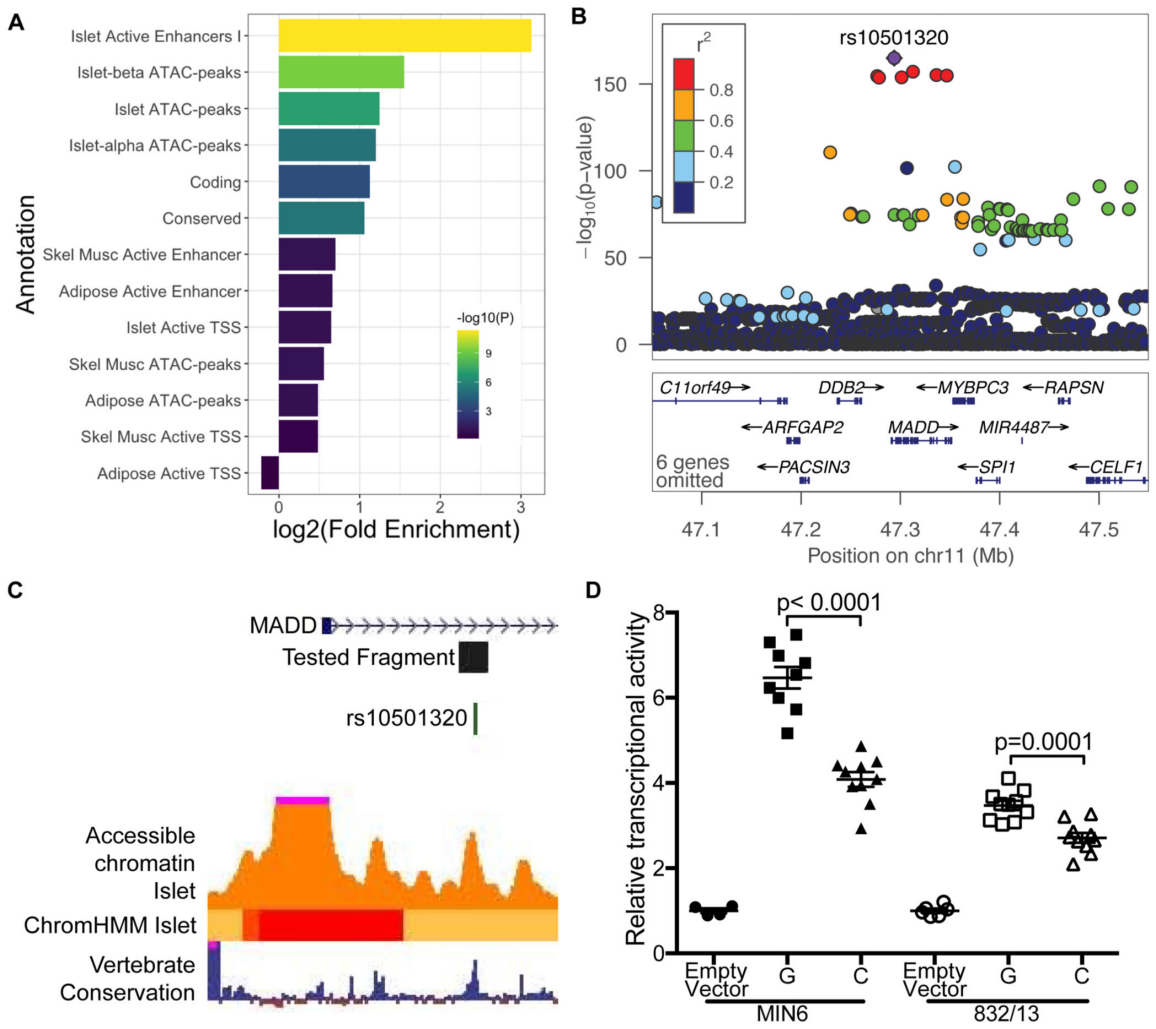
Candidate variants may influence regulatory activity. A) regulatory element enrichment analyses using enhancers, accessible chromatin, and other data from islets, skeletal muscle, and adipose. Proinsulin variants are enriched in islet active enhancers and accessible chromatin, especially in beta cells. B) The *MADD* locus in proinsulin, lead variant rs10501320. The *MADD* region is an area of extensive LD – the full locus is shown in Figure S3. C) The lead variant of the primary *MADD* signal is located in an intron of *MADD* and is in accessible chromatin in islets and an enhancer state and a region conserved across species. D) A 411-bp genomic element spanning the lead variant rs10501320 showed strong enhancer activity in a transcriptional reporter assay in two beta cell lines: MIN6 and 832/13. EV: empty vector; G/C: alleles at the lead variant rs10501320. In the eQTL and GWAS data, the G allele at rs10501320 that showed higher transcriptional activity showed higher *MADD* expression levels in islets and is associated with higher proinsulin.

Among the 1,090 variants in the extended credible sets for all signals, 62 overlapped with an active enhancer in islets and 76 overlapped with an islet cell type single-nucleus ATAC-seq peak (Table S18). We thus examined regulatory annotations of proinsulin-associated credible sets. The variants were enriched in islet active enhancers (Figure 3, fold enrichment = 8.8, p-value = 4.6 x10^-12^). Among islet single-nucleus ATAC-seq peaks, beta cell peaks were most enriched (fold enrichment = 2.9, p-value = 5.1 x10^-10^).

To further investigate plausible allelic effects of one variant located in an annotated ATAC-seq peak, we examined the regulatory function of lead variant rs10501320, at *MADD*, in transcriptional reporter assays. *MADD* is a well-documented proinsulin locus associated with proinsulin-to-insulin conversion^65^. Compared to a negative control, a genomic fragment spanning rs10501320 and the surrounding ATAC-seq peak showed ~3-fold increased transcriptional activity in rat insulinoma 832/13 cells and a ~4-fold increase in transcriptional activity in mouse insulinoma MIN6 cells, consistent with a role as an enhancer (Figure 3, Figure S9). The rs10501320-G allele showed 1.3 to 1.6-fold greater transcriptional activity than the C allele (p-value <0.0001); the G allele was associated with higher proinsulin in this GWAS meta-analysis and higher fasting glucose previously^27^. The direction of effect was consistent with the *MADD* nonsense mutation rs35233100, which has been predicted to cause a loss of function and was associated with decreased proinsulin (Figure S9). These data suggest that rs10501320 may contribute to allele-specific differences in *MADD* transcriptional activity in islets. The direction of effect was consistent with the *MADD* nonsense mutation rs35233100, which has been predicted to cause a loss of function and was associated with decreased proinsulin (Figure S9)^10^. These data suggest that rs10501320 may contribute to allele-specific differences in *MADD* transcriptional activity in islets and further suggest that *MADD* is a causal transcript at this multigene locus^10,66^.

## Discussion

These genetic analyses of circulating proinsulin levels, based on large GWAS meta-analyses, identified 36 signals at 30 loci. We identified 12 previously-reported proinsulin loci and 18 additional proinsulin loci. We replicate associations with low-frequency variants at *TBC1D30*, *SGSM2*, and *MADD*, loci that had previously been reported in an exome array analysis in a single cohort^10^. The only previously-described proinsulin locus that our study did not replicate was one reported as a cohort-specific signal near *SV2B* *(*p-value = 0.17)^11^. Characterization of these loci through eQTL colocalization, coding and regulatory annotation, and nearby gene function (Tables S11-S14) provided candidate genes that may influence insulin processing and secretion.

Understanding how glycemic trait signals influence proinsulin can help elucidate potential pathways by which the variants may ultimately influence T2D. We identified five plausible broad groups of encoded proteins: prohormone convertases, beta-cell transcription, G-protein modulators, regulation of cytoskeleton dynamics, and lysosomal maturation/endosome recycling (Tables S11, S14). In the first group we include genes *PCSK1* and *PCSK2* encoding the prohormone convertases PCSK1/3 and PSCK2 that are respectively responsible for cleaving the B-Chain and A-Chain from the C-Peptide during proinsulin processing to insulin. While targeted studies have implicated an association between genetic variants in *PCSK2* and glucose homeostasis and T2D^67^, the association had not yet reached significance in a GWAS with T2D or other glycemic traits, and one study had suggested that *PCSK2* did not significantly impact the beta cells’ ability to produce mature insulin^68^. We now demonstrate that the association reaches genome-wide significance in proinsulin, supporting a significant role for *PCSK2* in beta cells during the processing of proinsulin to insulin. The second group includes candidate genes implicated in beta-cell differentiation (*BARHL1* at the *DDX31* locus, *JARID2*, *NKX6-3*, *SIX2*, and *SIX3)* or the activation and maintenance of beta-cell transcription (*BCL11A*, *C2CD4B*, *TCF7L2*, and *TLE1*). For example, *JARID2* has been shown to play a role in pancreatic and endocrine cell differentiation and beta-cell mass in mouse embryos^69–71^. The third group consists of genes mediating vesicle translocation and membrane fusion events by affecting the activity of small G proteins, such as Rab and Rho GTPases. *DLC1*, at the *DLC1* locus, encodes a GTPase activating protein that promotes actin polymerization through regulating the Rho/Rock1 and is modulated by insulin-responsive pathways^72,73^. The three remaining loci in this group are established proinsulin loci whose nearby genes have been described previously (*MADD*, *SGSM2*, and *TBC1D30)*^10^. The fourth group is comprised of genes affecting the cytoskeleton, which undergoes dynamic changes during the processing and secretion of proinsulin at basal and stimulated states: *ANK1*, *KANK1*, *LRRC49*, and *RNF6*. *KANK1* promotes exocytotic events by mediating actin polymerization^74^; *LRRC49* at the *LARP6* locus is a member of the tubulin polyglutamylase complex^75^; and *RNF6* is an E3 ubiquitin-protein ligase that regulates actin remodeling^76,77^. Finally, the fifth group includes genes (*ELAPOR1*, *SNX7*, *STX16*, *TPD52*, *WIPI1*, and *ARSG*) implicated in endosome recycling and lysosomal maturation. In the beta cells, proinsulin is degraded in autophagosome-derived lysosomes via an endocytotic pathway that contributes to the tight regulation of insulin secretion and glucose homeostasis^78,79^. Both *SNX7* (encoding a sorting nexin^80^) and *WIPI1* (encoding a WD40 repeat protein) play a role in forming autophagosome and transiting autophagosome to early endosome^81,82^. *STX16* encodes a t-SNARE involved in secretory vesicle membrane fusion and endosome recycling in the Golgi^83,84^. These genes might help further elucidate the mechanisms for insulin synthesis, processing, and secretion.

Previously proposed clusters of T2D loci included two related to insulin deficiency that differed based on the direction of effect of the T2D risk allele on circulating proinsulin levels^6–9^. The allele associated with higher glucose was associated with higher proinsulin for half the lead variants, including all variants located near genes involved in beta-cell dysfunction and transcriptional regulation (Tables S10, S11, S14). For the remaining proinsulin loci, the alleles associated with higher glucose were associated with lower proinsulin; many of these variants are located near genes involved in cytoskeleton dynamics, lysosomal maturation, or endosome recycling (e.g. *WIPI1*, *ELAPOR1*, and *RNF6*). Thus, the directions of allelic effect on proinsulin relative to glucose can help distinguish between clusters of T2D loci^6–9^.

As another approach to identify potential causal genes, we integrated GWAS signals with islet eQTLs through colocalization and SMR analyses. This approach identified four potential candidate genes at three loci that that have not previously been reported in proinsulin or any of the T2D and glycemic studies: *SLC2A10*, *SLC7A14*, *WIPI1*, and *ARSG*. Loci that colocalized with eQTL signals of more than one gene, such as *SIX3* and *WIPI1*, could correspond to allelic effects on more than one gene, sequential effects, or effects on both genes for which only one gene is physiologically relevant to the trait. Our eQTL colocalization analyses also showed that the proinsulin signal at the *NKX6-3/ANK1* locus does not colocalize with the primary AGEN T2D signal and *NKX6-3* in islets, but rather with the secondary AGEN T2D signal and the *ANK1* eQTL in adipose^26,38,39^. Larger eQTL datasets and further characterization of their conditionally distinct signals may be valuable to better interpret colocalization with GWAS signals. Together, the several GWAS traits and eQTL colocalizations at this locus suggest that the underlying mechanisms are not yet fully understood. While we attempt to offer plausible candidate genes for all our proinsulin signals, the genes identified through physical proximity to the lead variant, coding variants in the credible set, islet expression, and literature searches (Table S11-14) are predictions; functional work is invaluable to elucidate genes’ roles in the proinsulin.

The *SIX3* proinsulin locus was described previously as a T2D and glucose signal in East Asians^26,27,85^. Both *SIX3* and *SIX2* are highly and specifically expressed in islets, with an iESI score of 10 for both genes. *SIX3* regulates beta cell development coordinately with *SIX2*, and knockdown of either gene impairs insulin secretion^86,87^. Despite a common allele frequency (MAF > 0.13 for all 1000 Genomes ancestries) across ancestries and evidence that the lead variant affects transcriptional factor binding and transcriptional activity^85^, GWAS meta-analyses of T2D and fasting glucose have failed to date to identify an association at p-value<5x10^-8^ in European-ancestry individuals^24,27^. Our proinsulin results demonstrate that the glycemic associations at this *SIX3* signal are not specific to East Asians (Figure S10).

The primary *STARD10* signal, which colocalized with a T2D^24–26^ signal, also colocalized with both the *STARD10* and *ARAP1* lead islet eQTL signals (Figure S11). The proinsulin-decreasing allele at the *STARD10* lead variant (rs77464186) was associated with decreased expression of both *STARD10* and *ARAP1*. Although the strength of association was stronger with *STARD10* expression (eQTL p-value with rs77464186 for *STARD10* expression = 5x10^-34^ vs. *ARAP1* expression = 6x10^-7^), the evidence for colocalization was stronger with *ARAP1* (*ARAP1* r^2^ = 0.99, Posterior Probability of Full Colocalization, PPFC = 0.9) vs. *STARD10* (r^2^ = 0.93, PPFC = 0.60). Both *STARD10* and *ARAP1* are highly expressed in islets, with iESI scores of 9 and 7, respectively. The strength and direction of association between proinsulin and *STARD10* were consistent with the evidence that *STARD10* influences insulin granule biosynthesis and insulin processing by binding to phosphatidylinositides; beta cell deletion of *Stard10* in mice led to impaired insulin secretion while overexpression of *Stard10* improved glucose tolerance in high fat-fed animals^88,89^.

Approximate conditional analysis software such as GCTA requires use of a large LD reference panel representative of the study participants. Even among single-ancestry analyses like this European-only proinsulin meta-analysis, use of different LD reference panels of the same broad European ancestry can result in strikingly different signals. This issue is particularly noticeable in regions with at least one strongly significant signal. For example, at the *MADD* locus (p = 1.4x10^-165^), GCTA analyses identified nine, twelve, or twenty-two conditionally distinct signals, depending on which reference panel we employed (Table S6). The discrepancy in results led us to report a signal only when we observed it in at least two of three reference panels, reducing the total number of signals in the *MADD* locus to three – all of which had been previously reported to be associated with proinsulin, adding further confidence to the validity of these signals. While identifying conditionally distinct signals using meta-analysis summary results is invaluable, caution in interpretation of signals is warranted.

To identify potential causal variants driving our observed signals that would have been missed in the regular credible sets built by the Bayesian fine-mapping approach from the association results alone, we defined an extended credible set as the union of variants in the Bayesian credible set and variants in high LD with the lead variant (r^2^ > 0.8 in 1000 Genomes European). This approach recognizes that standard fine-mapping approaches may be mis-calibrated when applied to meta-analyses^90^, and that variants may have been excluded from the meta-analysis due to analytic or technical factors (e.g. indels are not imputed by the Haplotype Reference Consortium or variants with MAF < 0.5%), as well as variants that were poorly represented in our meta-analysis due to factors such as low sample size. The extended credible set approach added 276 variants, including 142 variants that were not included in the meta-analysis and therefore could not have been included in the Bayesian credible set. The extended credible set identified an additional missense variant in *PCSK1* (rs6234), 15 variants that overlap active enhancers in islets, and 24 variants that overlap islet single nucleotide ATAC-seq cluster peaks. The extended credible sets provide a more comprehensive pool of candidate variants for mechanistic studies.

Integration of proinsulin loci with complementary glycemic traits, expression data in trait-relevant tissues, and functional follow-up provide candidate genes for T2D and hypotheses on potential avenues of mechanism for known T2D loci. While these proinsulin meta-analyses include a large sample size, the difficulty and cost in obtaining proinsulin measurements limits the sample size compared to studies of many other glycemic traits. Future research into genetic contributors to proinsulin will benefit from more and more diverse cohorts. Nonetheless, these findings may help accelerate our understanding of T2D disease pathology and promote translation into new therapeutics.

## Supplementary Material

Supplementary

## Figures and Tables

**Table 1:** Thirty loci associated with plasma proinsulin levels

Locus	rsid	Chr	Position	EA/NEA	EAF	Beta	StdErr	P-value
*SIX3*	rs12712928	2	45192080	C/G	0.16	0.09	0.01	1.5X10^-21^
*ELAPOR1*	rs74920406	1	109704525	C/T	0.96	0.15	0.02	3.7X10^-16^
*TLE1*	rs2796441	9	84308948	G/A	0.59	0.05	0.01	9.6X10^-14^
*TPD52*	rs1346146	8	81047278	T/C	0.45	0.05	0.01	2.0X10^-13^
*GIPR*	rs10423928	19	46182304	A/T	0.22	0.06	0.01	7.6X10^-12^
*STX16*	rs218473	20	57235980	C/T	0.32	0.05	0.01	1.5X10^-10^
*DLC1*	rs2977105	8	12794444	C/T	0.82	0.06	0.01	1.0X10^-09^
*FAM46C*	rs826415	1	118153977	T/G	0.67	0.04	0.01	1.3X10^-09^
*PCSK2*	rs111925767	20	17331621	T/G	0.23	0.05	0.01	1.6X10^-09^
*RNF6*	rs10507349	13	26781528	G/A	0.78	0.05	0.01	1.9X10^-09^
*PAM*	rs75457267	5	102658770	C/T	0.96	0.10	0.02	2.2X10^-09^
*SLC7A14*	rs56252324	3	170334547	A/C	0.87	0.06	0.01	5.4X10^-09^
*WIPI1*	rs2302783	17	66447073	C/T	0.72	0.04	0.01	1.1X10^-08^
*NKX6-3/ANK1*	rs13266210	8	41533514	G/A	0.21	0.05	0.01	2.1X10^-08^
*FAM185A*	rs10228495	7	102440184	C/T	0.45	0.04	0.01	2.9X10^-08^
*JARID2*	rs16876519	6	15496122	A/G	0.85	0.05	0.01	3.5X10^-08^
***Previously-reported loci***								
*STARD10*	rs77464186	11	72460398	C/A	0.19	0.26	0.01	3.7X10^-202^
*MADD*	rs10501320	11	47293799	G/C	0.76	0.21	0.01	1.3X10^-165^
*PCSK1*	rs13169290	5	95729406	A/G	0.28	0.12	0.01	3.3X10^-59^
*CDC4A/B*	rs11856307	15	62399093	A/C	0.54	0.09	0.01	6.4X10^-40^
*TCF7L2*	rs7903146	10	114758349	T/C	0.26	0.10	0.01	1.9X10^-39^
*SLC30A8*	rs4300038	8	118217915	G/A	0.66	0.09	0.01	4.1X10^-39^
*LARP6*	rs113350503	15	71111437	G/A	0.57	0.06	0.01	6.5X10^-18^
*DDX31*	rs368476	9	135456552	A/G	0.65	0.07	0.01	7.6X10^-21^
*SNX7*	rs6702126	1	99199954	G/A	0.65	0.04	0.01	8.7X10^-10^
*SGSM2*	rs61741902	17	2282779	A/G	0.01	0.47	0.03	5.8X10^-49^
*TBC1D30*	rs150781447	12	65224220	T/C	0.02	0.30	0.04	9.1X10^-17^
*KANK1*	rs146375546	9	727176	G/A	0.03	0.26	0.04	4.3X10^-11^
***Loci in model without BMI adjustment***
*SLC2A10*	rs3091537	20	45332200	A/C	0.64	0.04	0.01	3.9X10^-08^
*BCL11A*	rs243018	2	60586707	G/C	0.45	0.04	0.01	2.4X10^-08^

Chr, chromosome; EA, effect allele; NEA, non-effect allele; EAF, effect allele frequency; StdErr, standard error of beta. Loci are labeled by one or more nearby candidate genes.

**Table 2:** Six conditionally distinct proinsulin signals

	Marginal associations						Conditional associations			
Locus	rsid	EA/ NEA	EAF	Beta	StdErr	P-value	bC	bC_se	pC	LD with Primary (r^2^)
*STARD10*	rs481206	C/T	0.69	0.12	0.01	3.8X10^-62^	0.06	0.01	1.0X10^-16^	0.068
*MADD*	rs35233100	C/T	0.94	0.35	0.02	1.9X10^-104^	0.23	0.02	3.0X10^-46^	0.154
*MADD*	rs1449626	A/C	0.78	0.01	0.01	4.8X10^-01^	0.06	0.01	7.0X10^-15^	0.068
*PCSK1*	rs2117141	C/T	0.41	0.06	0.01	4.0X10^-16^	0.07	0.01	1.9X10^-24^	0.008
*SGSM2*	rs2447103	C/A	0.51	0.07	0.01	3.5X10^-26^	0.07	0.01	5.3X10^-22^	0.004
*DDX31*	rs7864386	G/A	0.56	0.03	0.01	1.6X10^-06^	0.04	0.01	1.8X10^-10^	0.027

Conditionally distinct signals identified using GCTA-COJO and the eMERGE reference panel. EA, effect allele; NEA, non-effect allele; EAF, effect allele frequency; StdErr, standard error of beta; bC, conditional beta; bC_se, conditional standard error of beta; pC, conditional p-value. Results for both *MADD* signals are from the analyses conditioning on the other two *MADD* signals.

## Data Availability

Upon publication, GWAS summary statistics will be available on the MAGIC Investigators website: https://magicinvestigators.org/downloads/ and through the Common Metabolic Diseases knowledge portal https://hugeamp.org/

## References

[1] Porte D (1991). Banting lecture 1990. Beta-cells in type II diabetes mellitus. Diabetes.

[2] Ward WK, Bolgiano DC, McKnight B, Halter JB, Porte D (1984). Diminished B cell secretory capacity in patients with noninsulin-dependent diabetes mellitus. J Clin Invest.

[3] Mezza T, Ferraro PM, Sun VA, Moffa S, Cefalo CMA, Quero G, Cinti F, Sorice GP, Pontecorvi A, Folli F (2018). Increased β-Cell Workload Modulates Proinsulin-to-Insulin Ratio in Humans. Diabetes.

[4] Liu M, Weiss MA, Arunagiri A, Yong J, Rege N, Sun J, Haataja L, Kaufman RJ, Arvan P (2018). Biosynthesis, structure, and folding of the insulin precursor protein. Diabetes, Obes Metab.

[5] Strawbridge RJ, Dupuis J, Prokopenko I, Barker A, Ahlqvist E, Rybin D, Petrie JR, Travers ME, Bouatia-Naji N, Dimas AS (2011). Genome-Wide Association Identifies Nine Common Variants Associated With Fasting Proinsulin Levels and Provides New Insights Into the Pathophysiology of Type 2 Diabetes. Diabetes.

[6] Udler MS, Kim J, von Grotthuss M, Bonàs-Guarch S, Cole JB, Chiou J, Boehnke M, Laakso M, Atzmon G, Glaser B (2018). Type 2 diabetes genetic loci informed by multi-trait associations point to disease mechanisms and subtypes: A soft clustering analysis. PLOS Med.

[7] Mansour Aly D, Dwivedi OP, Prasad RB, Käräjämäki A, Hjort R, Thangam M, Åkerlund M, Mahajan A, Udler MS, Florez JC (2021). Genome-wide association analyses highlight etiological differences underlying newly defined subtypes of diabetes. Nat Genet.

[8] DiCorpo D, LeClair J, Cole JB, Sarnowski C, Ahmadizar F, Bielak LF, Blokstra A, Bottinger EP, Chaker L, Chen Y-DI (2022). Type 2 Diabetes Partitioned Polygenic Scores Associate With Disease Outcomes in 454,193 Individuals Across 13 Cohorts. Diabetes Care.

[9] Wesolowska-Andersen A, Brorsson CA, Bizzotto R, Mari A, Tura A, Koivula R, Mahajan A, Vinuela A, Tajes JF, Sharma S (2022). Four groups of type 2 diabetes contribute to the etiological and clinical heterogeneity in newly diagnosed individuals: An IMI DIRECT study. Cell Reports Med.

[10] Huyghe JR, Jackson AU, Fogarty MP, Buchkovich ML, Stančáková A, Stringham HM, Sim X, Yang L, Fuchsberger C, Cederberg H (2013). Exome array analysis identifies new loci and low-frequency variants influencing insulin processing and secretion. Nat Genet.

[11] Strawbridge RJ, Silveira A, den Hoed M, Gustafsson S, Luan J, Rybin D, Dupuis J, Li-Gao R, Kavousi M, Dehghan A (2017). Identification of a novel proinsulin-associated SNP and demonstration that proinsulin is unlikely to be a causal factor in subclinical vascular remodelling using Mendelian randomisation. Atherosclerosis.

[12] Auton A, Abecasis GR, Altshuler DM, Durbin RM, Abecasis GR, Bentley DR, Chakravarti A, Clark AG, Donnelly P, Eichler EE (2015). A global reference for human genetic variation. Nature.

[13] Chien L-C (2020). A rank-based normalization method with the fully adjusted full-stage procedure in genetic association studies. PLoS One.

[14] Kang HM, Sul JH, Service SK, Zaitlen NA, Kong S, Freimer NB, Sabatti C, Eskin E (2010). Variance component model to account for sample structure in genome-wide association studies. Nat Genet.

[15] Zhan X, Hu Y, Li B, Abecasis GR, Liu DJ (2016). RVTESTS: An efficient and comprehensive tool for rare variant association analysis using sequence data. Bioinformatics.

[16] Purcell S, Neale B, Todd-Brown K, Thomas L, Ferreira MAR, Bender D, Maller J, Sklar P, de Bakker PIW, Daly MJ (2007). PLINK: A Tool Set for Whole-Genome Association and Population-Based Linkage Analyses. Am J Hum Genet.

[17] Winkler TW, Day FR, Croteau-Chonka DC, Wood AR, Locke AE, Mägi R, Ferreira T, Fall T, Graff M, Justice AE (2014). Quality control and conduct of genome-wide association meta-analyses. Nat Protoc.

[18] Willer CJ, Li Y, Abecasis GR (2010). METAL: fast and efficient meta-analysis of genomewide association scans. Bioinformatics.

[19] Yang J, Lee SH, Goddard ME, Visscher PM (2011). GCTA: a tool for genome-wide complex trait analysis. Am J Hum Genet.

[20] Yang J, Ferreira T, Morris AP, Medland SE, Madden PAF, Heath AC, Martin NG, Montgomery GW, Genetic Investigation of ANthropometric Traits (GIANT) Consortium, DIAbetes Genetics Replication And Meta-analysis (DIAGRAM) Consortium (2012). Conditional and joint multiple-SNP analysis of GWAS summary statistics identifies additional variants influencing complex traits. Nat Genet.

[21] Stancakova A, Paananen J, Soininen P, Kangas AJ, Bonnycastle LL, Morken MA, Collins FS, Jackson AU, Boehnke ML, Kuusisto J (2011). Effects of 34 Risk Loci for Type 2 Diabetes or Hyperglycemia on Lipoprotein Subclasses and Their Composition in 6,580 Nondiabetic Finnish Men. Diabetes.

[22] Rolfe EDL, Loos RJF, Druet C, Stolk RP, Ekelund U, Griffin SJ, Forouhi NG, Wareham NJ, Ong KK (2010). Association between birth weight and visceral fat in adults. Am J Clin Nutr.

[23] McCarty CA, Chisholm RL, Chute CG, Kullo IJ, Jarvik GP, Larson EB, Li R, Masys DR, Ritchie MD, Roden DM (2011). The eMERGE Network: A consortium of biorepositories linked to electronic medical records data for conducting genomic studies. BMC Med Genomics.

[24] Mahajan A, Taliun D, Thurner M, Robertson NR, Torres JM, Rayner NW, Payne AJ, Steinthorsdottir V, Scott RA, Grarup N (2018). Fine-mapping type 2 diabetes loci to single-variant resolution using high-density imputation and islet-specific epigenome maps. Nat Genet.

[25] Mahajan A, Spracklen CN, Zhang W, Ng MCY, Petty LE, Kitajima H, Yu GZ, Rüeger S, Speidel L, Kim YJ (2022). Multi-ancestry genetic study of type 2diabetes highlights the power of diverse populations for discovery and translation. Nat Genet.

[26] Spracklen CN, Horikoshi M, Kim YJ, Lin K, Bragg F, Moon S, Suzuki K, Tam CHT, Tabara Y, Kwak S-H (2020). Identification of type 2 diabetes loci in 433,540 East Asian individuals. Nature.

[27] Chen J, Spracklen CN, Marenne G, Varshney A, Corbin LJ, Luan J, Willems SM, Wu Y, Zhang X, Horikoshi M (2021). The trans-ancestral genomic architecture of glycemic traits. Nat Genet.

[28] Foley CN, Staley JR, Breen PG, Sun BB, Kirk PDW, Burgess S, Howson JMM (2021). A fast and efficient colocalization algorithm for identifying shared genetic risk factors across multiple traits. Nat Commun.

[29] Giambartolomei C, Vukcevic D, Schadt EE, Franke L, Hingorani AD, Wallace C, Plagnol V (2014). Bayesian test for colocalisation between pairs of genetic association studies using summary statistics. PLoS Genet.

[30] Carey V (2021). ldblock:data structures for linkage disequilibrium measures in populations. R Packag.

[31] Yin X, Chan LS, Bose D, Jackson AU, VandeHaar P, Locke AE, Fuchsberger C, Stringham HM, Welch R, Yu K (2022). Genome-wide association studies of metabolites in Finnish men identify disease-relevant loci. Nat Commun.

[32] Ghoussaini M, Mountjoy E, Carmona M, Peat G, Schmidt EM, Hercules A, Fumis L, Miranda A, Carvalho-Silva D, Buniello A (2021). Open Targets Genetics: systematic identification of trait-associated genes using large-scale genetics and functional genomics. Nucleic Acids Res.

[33] Mountjoy E, Schmidt EM, Carmona M, Schwartzentruber J, Peat G, Miranda A, Fumis L, Hayhurst J, Buniello A, Karim MA (2021). An open approach to systematically prioritize causal variants and genes at all published human GWAS trait-associated loci. Nat Genet.

[34] Buniello A, MacArthur JAL, Cerezo M, Harris LW, Hayhurst J, Malangone C, McMahon A, Morales J, Mountjoy E, Sollis E (2019). The NHGRI-EBI GWAS Catalog of published genome-wide association studies, targeted arrays and summary statistics 2019. Nucleic Acids Res.

[35] Zhou W, Nielsen JB, Fritsche LG, Dey R, Gabrielsen ME, Wolford BN, LeFaive J, VandeHaar P, Gagliano SA, Gifford A (2018). Efficiently controlling for case-control imbalance and sample relatedness in large-scale genetic association studies. Nat Genet.

[36] Kurki MI, Karjalainen J, Palta P, Sipilä TP, Kristiansson K, Donner K, Reeve MP, Laivuori H, Aavikko M, Kaunisto MA (2022). FinnGen: Unique genetic insights from combining isolated population and national health register data. MedRxiv.

[37] Varshney A, Scott LJ, Welch RP, Erdos MR, Chines PS, Narisu N, Albanus RD, Orchard P, Wolford BN, Kursawe R (2017). Genetic regulatory signatures underlying islet gene expression and type 2 diabetes. Proc Natl Acad Sci.

[38] Viñuela A, Varshney A, van de Bunt M, Prasad RB, Asplund O, Bennett A, Boehnke M, Brown AA, Erdos MR, Fadista J (2020). Genetic variant effects on gene expression in human pancreatic islets and their implications for T2D. Nat Commun.

[39] Raulerson CK, Ko A, Kidd JC, Currin KW, Brotman SM, Cannon ME, Wu Y, Spracklen CN, Jackson AU, Stringham HM (2019). Adipose Tissue Gene Expression Associations Reveal Hundreds of Candidate Genes for Cardiometabolic Traits. Am J Hum Genet.

[40] Pruim RJ, Welch RP, Sanna S, Teslovich TM, Chines PS, Gliedt TP, Boehnke M, Abecasis GR, Willer CJ, Frishman D (2010). LocusZoom: Regional visualization of genome-wide association scan results. Bioinformatics.

[41] Zhu Z, Zhang F, Hu H, Bakshi A, Robinson MR, Powell JE, Montgomery GW, Goddard ME, Wray NR, Visscher PM (2016). Integration of summary data from GWAS and eQTL studies predicts complex trait gene targets. Nat Genet.

[42] Maller JB, McVean G, Byrnes J, Vukcevic D, Palin K, Su Z, Howson JMM, Auton A, Myers S, Wellcome Trust Case Control Consortium (2012). Bayesian refinement of association signals for 14 loci in 3 common diseases. Nat Genet.

[43] Neph S, Kuehn MS, Reynolds AP, Haugen E, Thurman RE, Johnson AK, Rynes E, Maurano MT, Vierstra J, Thomas S (2012). BEDOPS: high-performance genomic feature operations. Bioinformatics.

[44] McLaren W, Pritchard B, Rios D, Chen Y, Flicek P, Cunningham F (2010). Deriving the consequences of genomic variants with the Ensembl API and SNP Effect Predictor. Bioinformatics.

[45] Ng PC, Henikoff S (2003). SIFT: Predicting amino acid changes that affect protein function. Nucleic Acids Res.

[46] Adzhubei IA, Schmidt S, Peshkin L, Ramensky VE, Gerasimova A, Bork P, Kondrashov AS, Sunyaev SR (2010). A method and server for predicting damaging missense mutations. Nat Methods.

[47] Rentzsch P, Schubach M, Shendure J, Kircher M (2021). CADD-Splice-improving genome-wide variant effect prediction using deep learning-derived splice scores. Genome Med.

[48] Kircher M, Witten DM, Jain P, O’Roak BJ, Cooper GM, Shendure J (2014). A general framework for estimating the relative pathogenicity of human genetic variants. Nat Genet.

[49] Reva B, Antipin Y, Sander C (2011). Predicting the functional impact of protein mutations: application to cancer genomics. Nucleic Acids Res.

[50] Varshney A, Kyono Y, Elangovan VR, Wang C, Erdos MR, Narisu N, Albanus RD, Orchard P, Stitzel ML, Collins FS (2021). A Transcription Start Site Map in Human Pancreatic Islets Reveals Functional Regulatory Signatures. Diabetes.

[51] Cannon ME, Currin KW, Young KL, Perrin HJ, Vadlamudi S, Safi A, Song L, Wu Y, Wabitsch M, Laakso M (2019). Open Chromatin Profiling in Adipose Tissue Marks Genomic Regions with Functional Roles in Cardiometabolic Traits. G3 (Bethesda).

[52] Rai V, Quang DX, Erdos MR, Cusanovich DA, Daza RM, Narisu N, Zou LS, Didion JP, Guan Y, Shendure J (2020). Single-cell ATAC-Seq in human pancreatic islets and deep learning upscaling of rare cells reveals cell-specific type 2 diabetes regulatory signatures. Mol Metab.

[53] Miguel-Escalada I, Bonàs-Guarch S, Cebola I, Ponsa-Cobas J, Mendieta-Esteban J, Atla G, Javierre BM, Rolando DMY, Farabella I, Morgan CC (2019). Human pancreatic islet three-dimensional chromatin architecture provides insights into the genetics of type 2 diabetes. Nat Genet.

[54] Schmidt EM, Zhang J, Zhou W, Chen J, Mohlke KL, Chen YE, Willer CJ (2015). GREGOR: evaluating global enrichment of trait-associated variants in epigenomic features using a systematic, data-driven approach. Bioinformatics.

[55] Fogarty MP, Cannon ME, Vadlamudi S, Gaulton KJ, Mohlke KL (2014). Identification of a regulatory variant that binds FOXA1 and FOXA2 at the CDC123/CAMK1D type 2 diabetes GWAS locus. PLoS Genet.

[56] Imai A, Ishida M, Fukuda M, Nashida T, Shimomura H (2013). MADD/DENN/Rab3GEP functions as a guanine nucleotide exchange factor for Rab27 during granule exocytosis of rat parotid acinar cells. Arch Biochem Biophys.

[57] Bailyes EM, Shennan KI, Seal AJ, Smeekens SP, Steiner DF, Hutton JC, Docherty K (1992). A member of the eukaryotic subtilisin family (PC3) has the enzymic properties of the type 1 proinsulin-converting endopeptidase. Biochem J.

[58] Davidson HW, Rhodes CJ, Hutton JC (1988). Intraorganellar calcium and pH control proinsulin cleavage in the pancreatic beta cell via two distinct site-specific endopeptidases. Nature.

[59] Scott LJ, Erdos MR, Huyghe JR, Welch RP, Beck AT, Wolford BN, Chines PS, Didion JP, Narisu N, Stringham HM (2016). The genetic regulatory signature of type 2 diabetes in human skeletal muscle. Nat Commun.

[60] Klimentidis YC, Arora A, Newell M, Zhou J, Ordovas JM, Renquist BJ, Wood AC (2020). Phenotypic and Genetic Characterization of Lower LDL Cholesterol and Increased Type 2 Diabetes Risk in the UK Biobank. Diabetes.

[61] Garber M, Guttman M, Clamp M, Zody MC, Friedman N, Xie X (2009). Identifying novel constrained elements by exploiting biased substitution patterns. Bioinformatics.

[62] Huber CD, Kim BY, Lohmueller KE (2020). Population genetic models of GERP scores suggest pervasive turnover of constrained sites across mammalian evolution. PLOS Genet.

[63] Ansarullah, Jain C, Far FF, Homberg S, Wißmiller K, von Hahn FG, Raducanu A, Schirge S, Sterr M, Bilekova S (2021). Inceptor counteracts insulin signalling in β-cells to control glycaemia. Nature.

[64] Møller AB, Kampmann U, Hedegaard J, Thorsen K, Nordentoft I, Vendelbo MH, Møller N, Jessen N (2017). Altered gene expression and repressed markers of autophagy in skeletal muscle of insulin resistant patients with type 2 diabetes. Sci Rep.

[65] Wagner R, Dudziak K, Herzberg-Schäfer SA, Machicao F, Stefan N, Staiger H, Häring H-U, Fritsche A (2011). Glucose-raising genetic variants in MADD and ADCY5 impair conversion of proinsulin to insulin. PLoS One.

[66] Cornes BK, Brody JA, Nikpoor N, Morrison AC, Chu H, Ahn BS, Wang S, Dauriz M, Barzilay JI, Dupuis J (2014). Association of levels of fasting glucose and insulin with rare variants at the chromosome 11p11.2-MADD locus: Cohorts for Heart and Aging Research in Genomic Epidemiology (CHARGE) Consortium Targeted Sequencing Study. Circ Cardiovasc Genet.

[67] Chang T-J, Chiu Y-F, Sheu WH-H, Shih K-C, Hwu C-M, Quertermous T, Jou Y-S, Kuo S-S, Chang Y-C, Chuang L-M (2015). Genetic polymorphisms of PCSK2 are associated with glucose homeostasis and progression to type 2 diabetes in a Chinese population. Sci Rep.

[68] Ramzy A, Asadi A, Kieffer TJ (2020). Revisiting Proinsulin Processing: Evidence That Human β-Cells Process Proinsulin With Prohormone Convertase (PC) 1/3 but Not PC2. Diabetes.

[69] Cervantes S, Fontcuberta-PiSunyer M, Servitja J-M, Fernandez-Ruiz R, García A, Sanchez L, Lee Y-S, Gomis R, Gasa R (2017). Late-stage differentiation of embryonic pancreatic β-cells requires Jarid2. Sci Rep.

[70] Soyer J, Flasse L, Raffelsberger W, Beucher A, Orvain C, Peers B, Ravassard P, Vermot J, Voz ML, Mellitzer G (2010). Rfx6 is an Ngn3-dependent winged helix transcription factor required for pancreatic islet cell development. Development.

[71] White P, Lee May C, Lamounier RN, Brestelli JE, Kaestner KH (2008). Defining Pancreatic Endocrine Precursors and Their Descendants. Diabetes.

[72] Liao Y-C, Lo SH (2008). Deleted in liver cancer-1 (DLC-1): a tumor suppressor not just for liver. Int J Biochem Cell Biol.

[73] Hers I, Wherlock M, Homma Y, Yagisawa H, Tavaré JM (2006). Identification of p122RhoGAP (deleted in liver cancer-1) Serine 322 as a substrate for protein kinase B and ribosomal S6 kinase in insulin-stimulated cells. J Biol Chem.

[74] Rafiq NBM, Nishimura Y, Plotnikov SV, Thiagarajan V, Zhang Z, Shi S, Natarajan M, Viasnoff V, Kanchanawong P, Jones GE (2019). A mechano-signalling network linking microtubules, myosin IIA filaments and integrin-based adhesions. Nat Mater.

[75] Wang L, Paudyal SC, Kang Y, Owa M, Liang F-X, Spektor A, Knaut H, Sánchez I, Dynlacht BD (2022). Regulators of tubulin polyglutamylation control nuclear shape and cilium disassembly by balancing microtubule and actin assembly. Cell Res.

[76] Liu L, Zhang Y, Wong CC, Zhang J, Dong Y, Li X, Kang W, Chan FKL, Sung JJY, Yu J (2018). RNF6 Promotes Colorectal Cancer by Activating the Wnt/β-Catenin Pathway via Ubiquitination of TLE3. Cancer Res.

[77] Tursun B, Schlüter A, Peters MA, Viehweger B, Ostendorff HP, Soosairajah J, Drung A, Bossenz M, Johnsen SA, Schweizer M (2005). The ubiquitin ligase Rnf6 regulates local LIM kinase 1 levels in axonal growth cones. Genes Dev.

[78] Riahi Y, Wikstrom JD, Bachar-Wikstrom E, Polin N, Zucker H, Lee M-S, Quan W, Haataja L, Liu M, Arvan P (2016). Autophagy is a major regulator of beta cell insulin homeostasis. Diabetologia.

[79] Zhou Y, Liu Z, Zhang S, Zhuang R, Liu H, Liu X, Qiu X, Zhang M, Zheng Y, Li L (2020). RILP Restricts Insulin Secretion Through Mediating Lysosomal Degradation of Proinsulin. Diabetes.

[80] Antón Z, Betin VMS, Simonetti B, Traer CJ, Attar N, Cullen PJ, Lane JD (2020). A heterodimeric SNX4--SNX7 SNX-BAR autophagy complex coordinates ATG9A trafficking for efficient autophagosome assembly. J Cell Sci.

[81] Velikkakath AKG, Nishimura T, Oita E, Ishihara N, Mizushima N (2012). Mammalian Atg2 proteins are essential for autophagosome formation and important for regulation of size and distribution of lipid droplets. Mol Biol Cell.

[82] Tsuyuki S, Takabayashi M, Kawazu M, Kudo K, Watanabe A, Nagata Y, Kusama Y, Yoshida K (2014). Detection of WIPI1 mRNA as an indicator of autophagosome formation. Autophagy.

[83] Hastoy B, Clark A, Rorsman P, Lang J (2017). Fusion pore in exocytosis: More than an exit gate? A β-cell perspective. Cell Calcium.

[84] Mallard F, Tang BL, Galli T, Tenza D, Saint-Pol A, Yue X, Antony C, Hong W, Goud B, Johannes L (2002). Early/recycling endosomes-to-TGN transport involves two SNARE complexes and a Rab6 isoform. J Cell Biol.

[85] Spracklen CN, Shi J, Vadlamudi S, Wu Y, Zou M, Raulerson CK, Davis JP, Zeynalzadeh M, Jackson K, Yuan W (2018). Identification and functional analysis of glycemic trait loci in the China Health and Nutrition Survey. PLoS Genet.

[86] Velazco-Cruz L, Goedegebuure MM, Maxwell KG, Augsornworawat P, Hogrebe NJ, Millman JR (2020). SIX2 Regulates Human β Cell Differentiation from Stem Cells and Functional Maturation In Vitro. Cell Rep.

[87] Bevacqua R, Lam J, Peiris H, Whitener R, Kim S, Gu X, Friedlander MSH, Kim SK (2021). SIX2 and SIX3 coordinately regulate functional maturity and fate of human pancreatic β cells. Genes Dev.

[88] Carrat GR, Haythorne E, Tomas A, Haataja L, Müller A, Arvan P, Piunti A, Cheng K, Huang M, Pullen TJ (2020). The type 2 diabetes gene product STARD10 is a phosphoinositide-binding protein that controls insulin secretory granule biogenesis. Mol Metab.

[89] Carrat GR, Hu M, Nguyen-Tu M-S, Chabosseau P, Gaulton KJ, van de Bunt M, Siddiq A, Falchi M, Thurner M, Canouil M (2017). Decreased STARD10 Expression Is Associated with Defective Insulin Secretion in Humans and Mice. Am J Hum Genet.

[90] Kanai M, Elzur R, Zhou W, Zhou W, Kanai M, Wu K-HH, Rasheed H, Tsuo K, Hirbo JB, Wang Y (2022). Meta-analysis fine-mapping is often miscalibrated at single-variant resolution. Cell Genomics.

