# Supplementary material for: Loci for insulin processing and secretion provide insight into type 2 diabetes risk
Source: Am J Hum Genet. Author manuscript; Available in PMC 2023 Jun 28. (PMC9943750; doi:10.1016/j.ajhg.2023.01.002)
Supplement: Supplementary [file EMS177405-supplement-Supplementary.pdf]

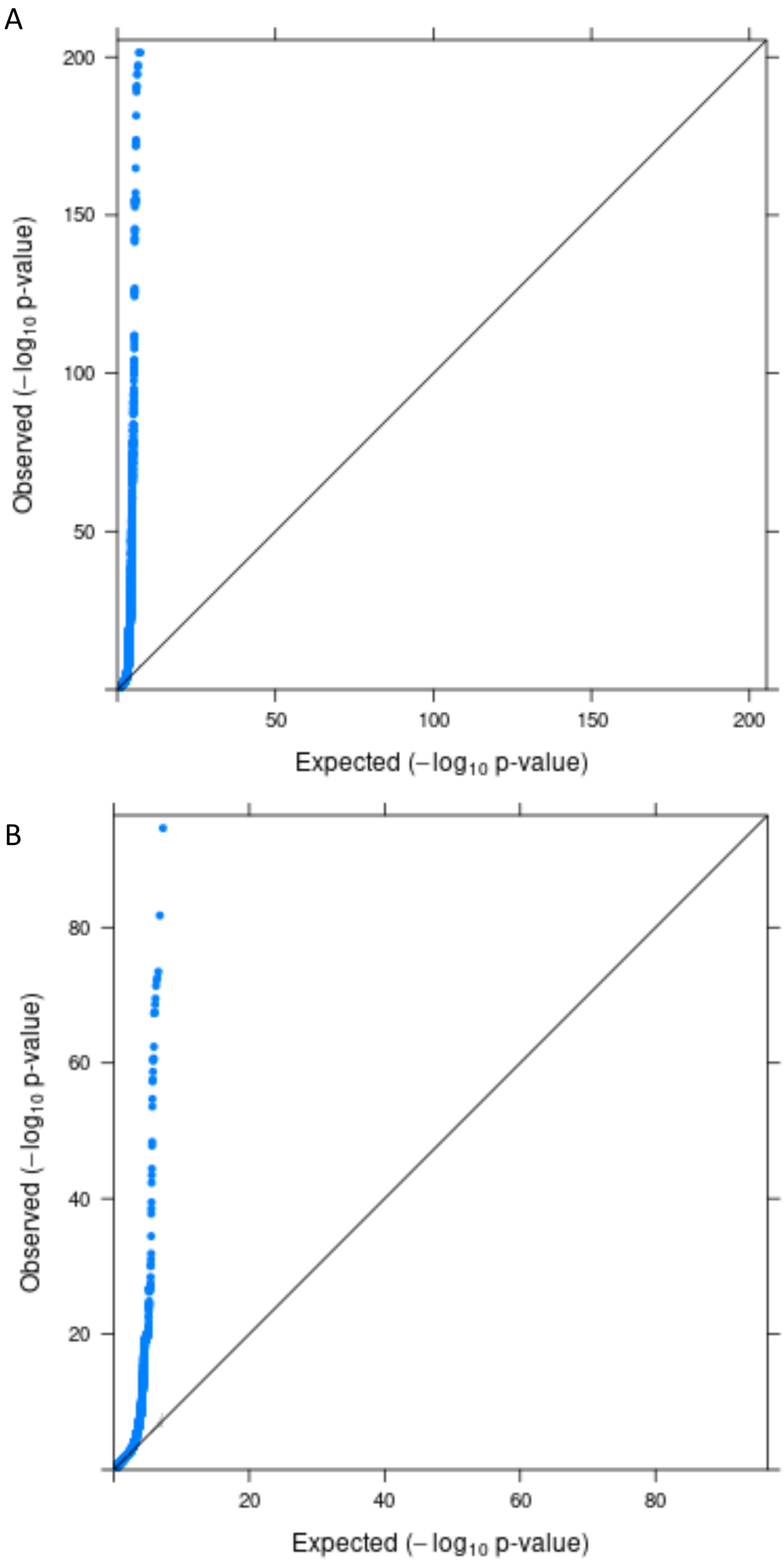

Figure S1: Quantile-quantile plots of the associations between variants and proinsulin adjusted for BMI. Panel A shows $-\log_{10}$(p-values) of all analyzed variants adjusted for BMI (X axis). Panel B shows $-\log_{10}$(p-values) after excluding all variants within 500 kb of a previously-identified signal.

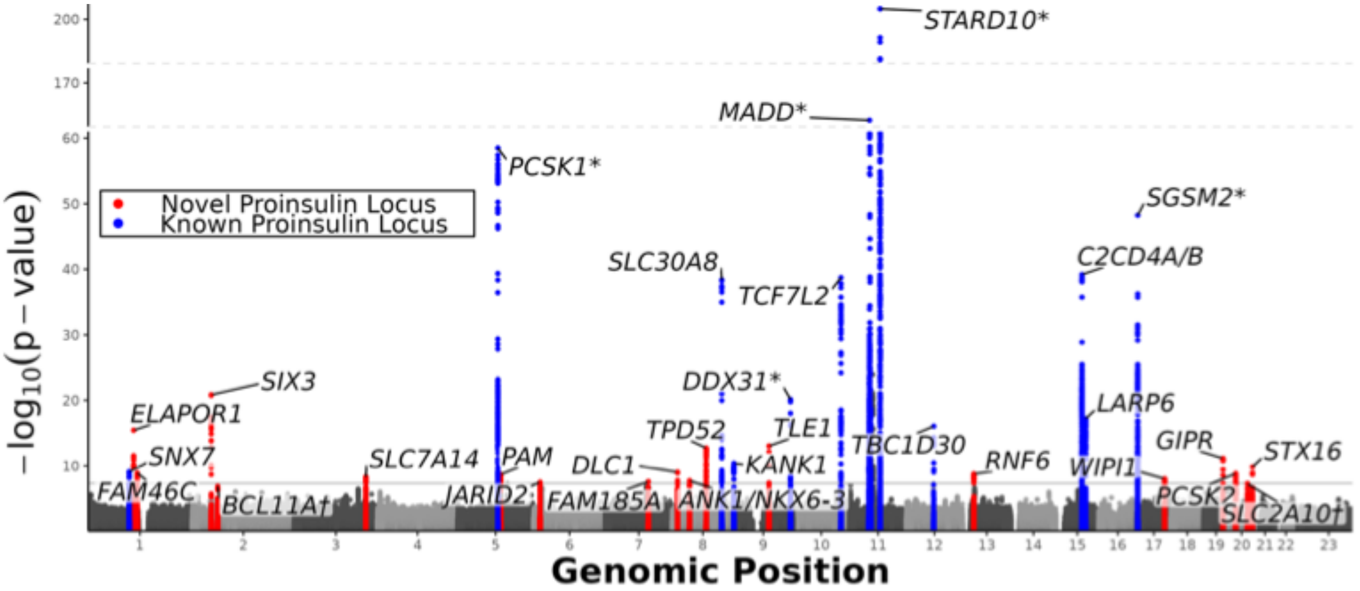

Figure S2: Manhattan plots of the associations between variants and proinsulin adjusted for BMI. Variants within 500 kb of a previously-known proinsulin locus are colored in blue; variants within 500 kb of a new proinsulin locus ($p<5x10^{-8}$) are colored in red. Loci with asterisks represent multi-signal loci. Loci with dagger represent loci only identified in models not adjusted for BMI.

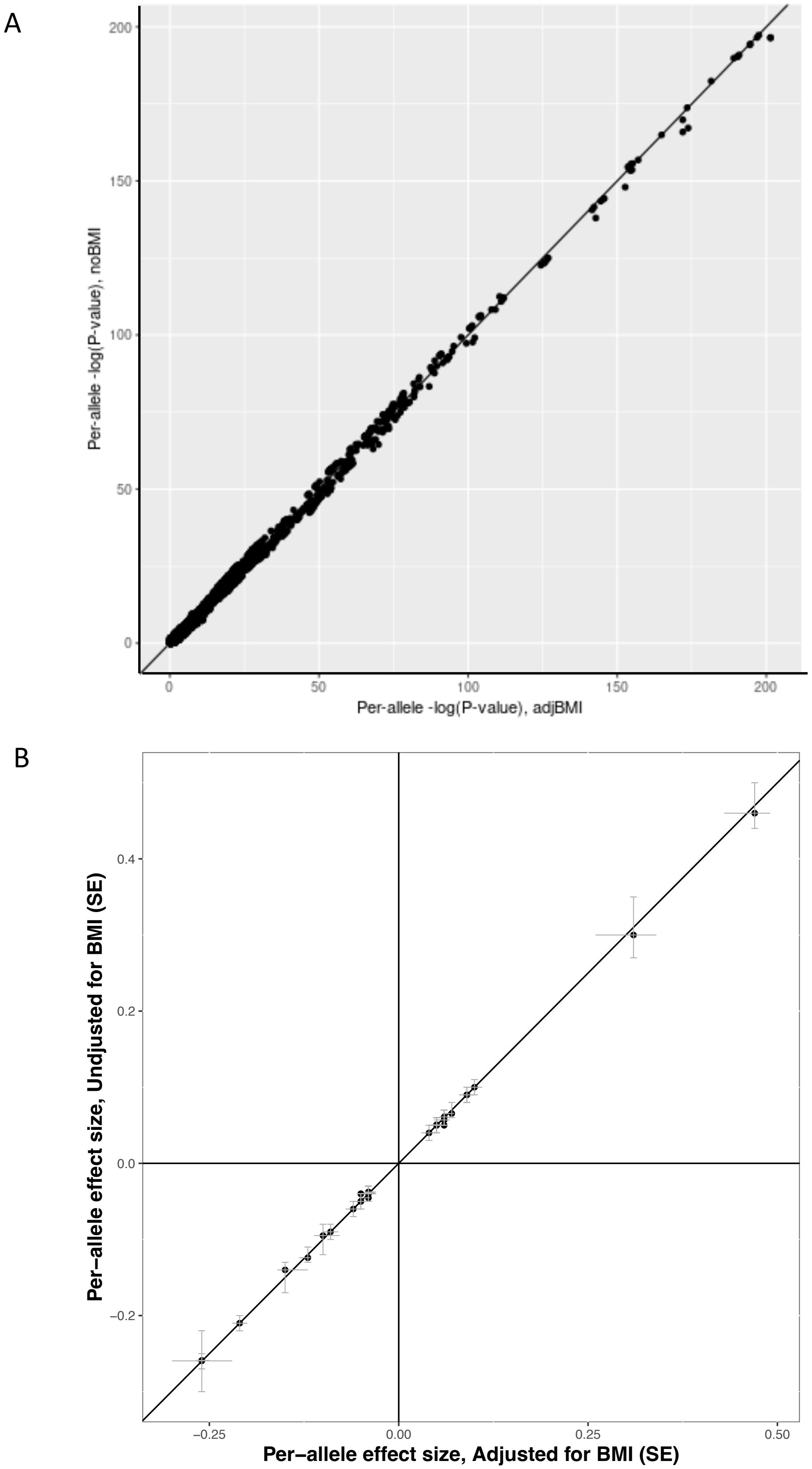

Figure S3: Influence of BMI on genetic associations with fasting proinsulin adjusting for BMI. Adjusting for BMI does little to influence the association between genetic variant and fasting proinsulin (Pearson Correlation, betas = 0.97). Panel A shows $-\log_{10}$(p-values) of all analyzed variants adjusted for BMI (X axis) against unadjusted for BMI (Y axis). Panel B shows the effect size (SE) of the significant lead variants with and without adjusting for BMI.

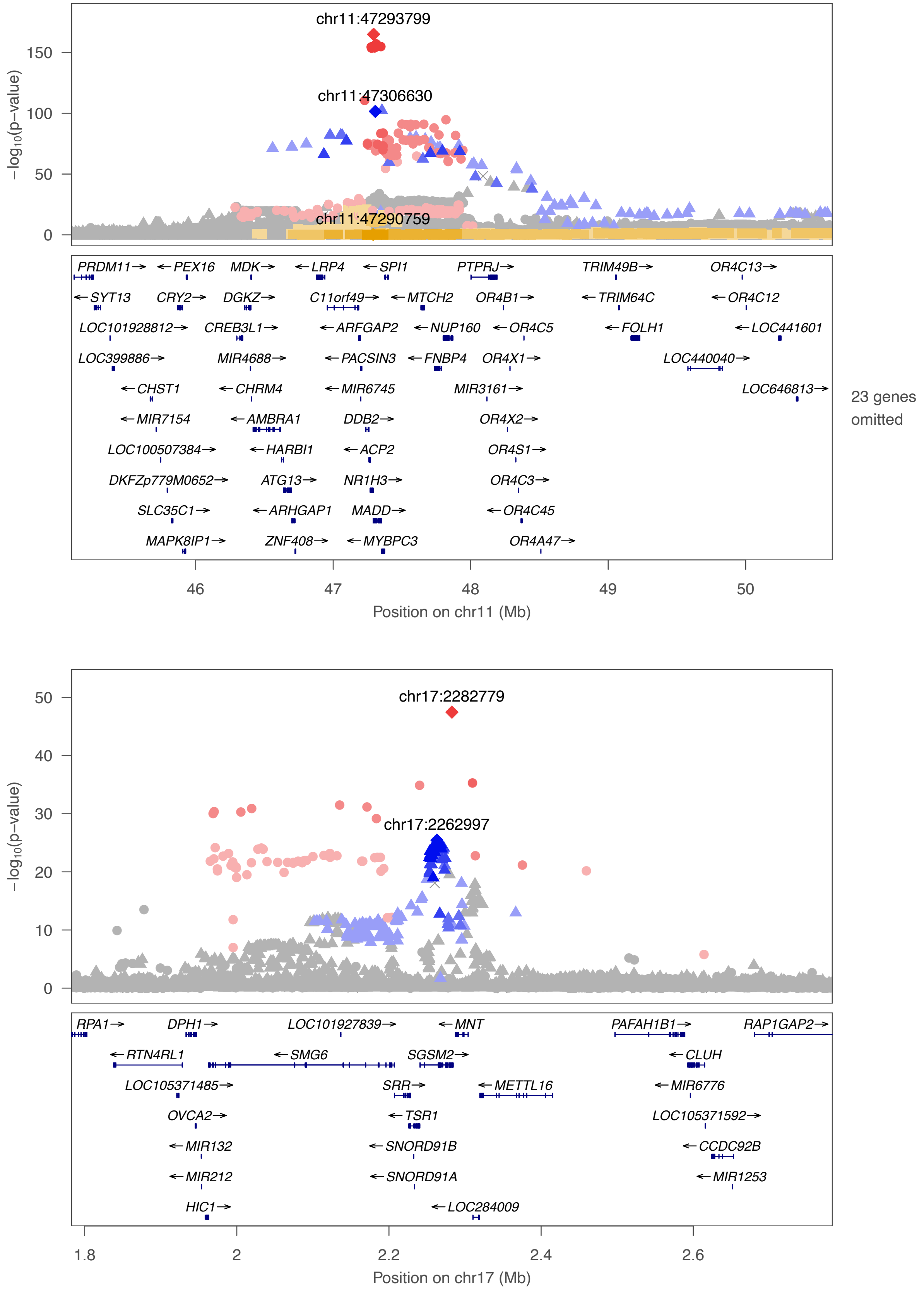

Figure S4: **Conditionally distinct signals in four multiple-signal loci**. Top, three signals in *MADD*. Bottom, two signals in *SGSM2.* The primary signal for the locus is shown in red, the secondary signal for the locus is shown in blue, and the tertiary locus is shown in yellow. Continued on the next page.

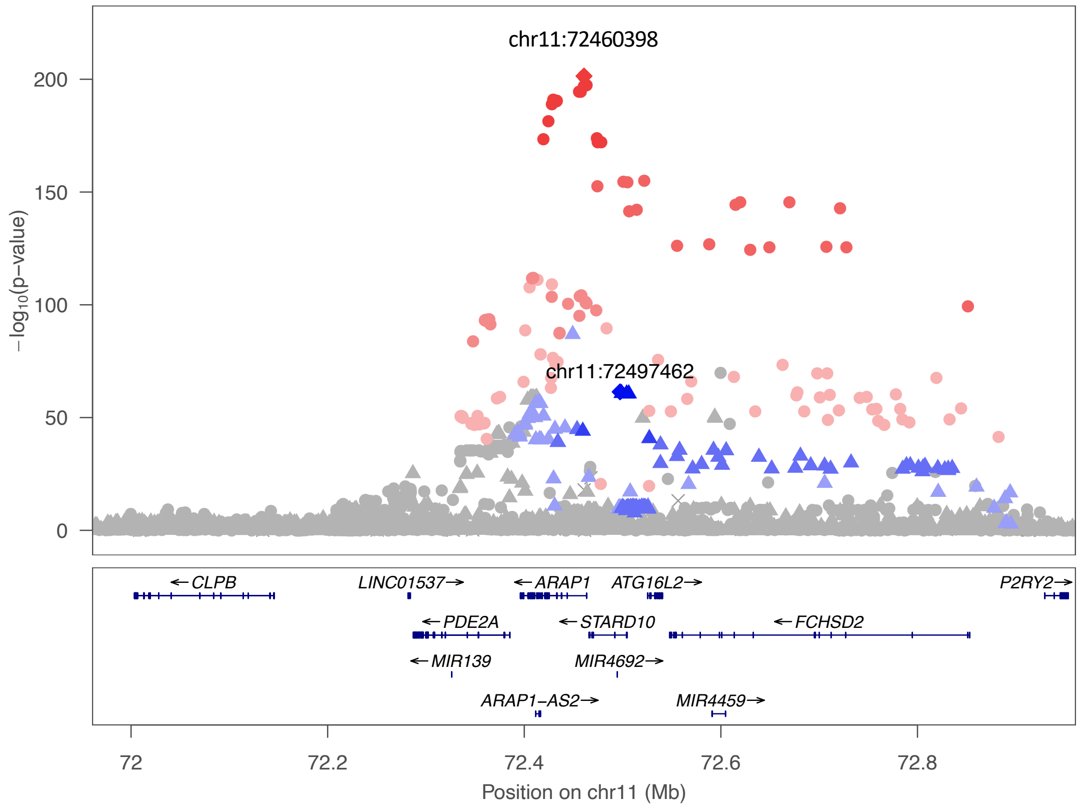

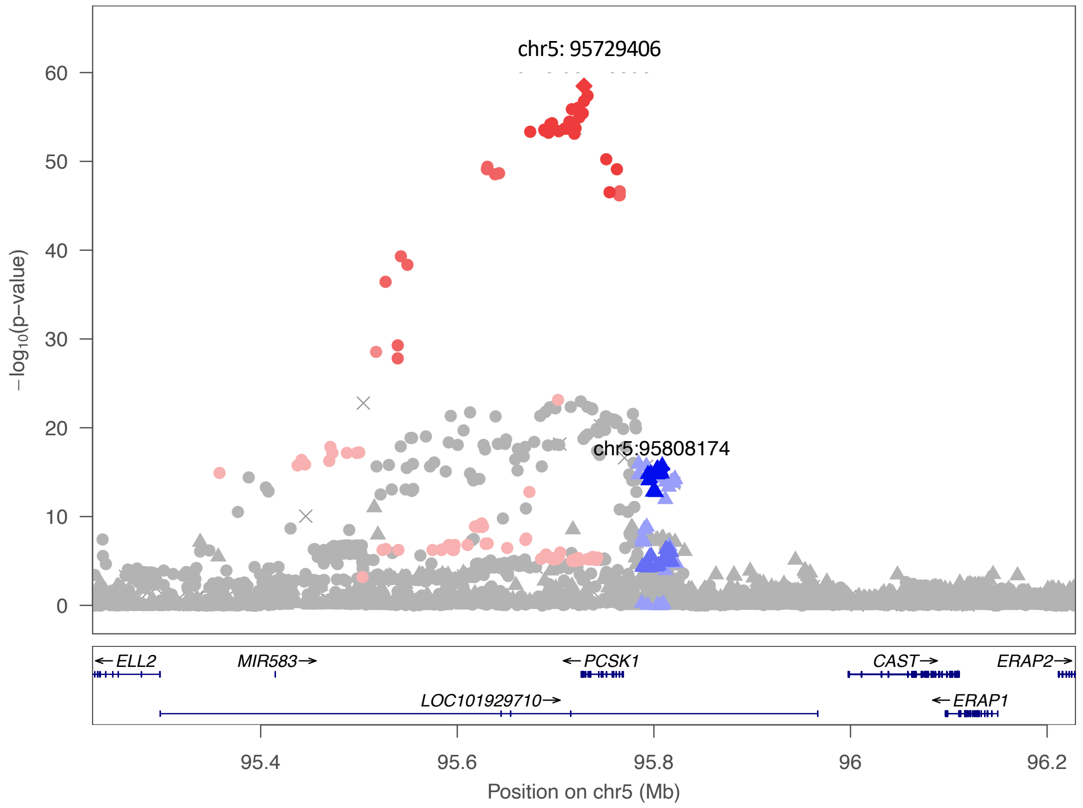

Figure S4 (continued): **Conditionally distinct signals in four multiple-signal loci**. Top, two signals in *STARD10*. Bottom, two signals in *PCSK1*. The primary signal for the locus is shown in red, the secondary signal for the locus is shown in blue.

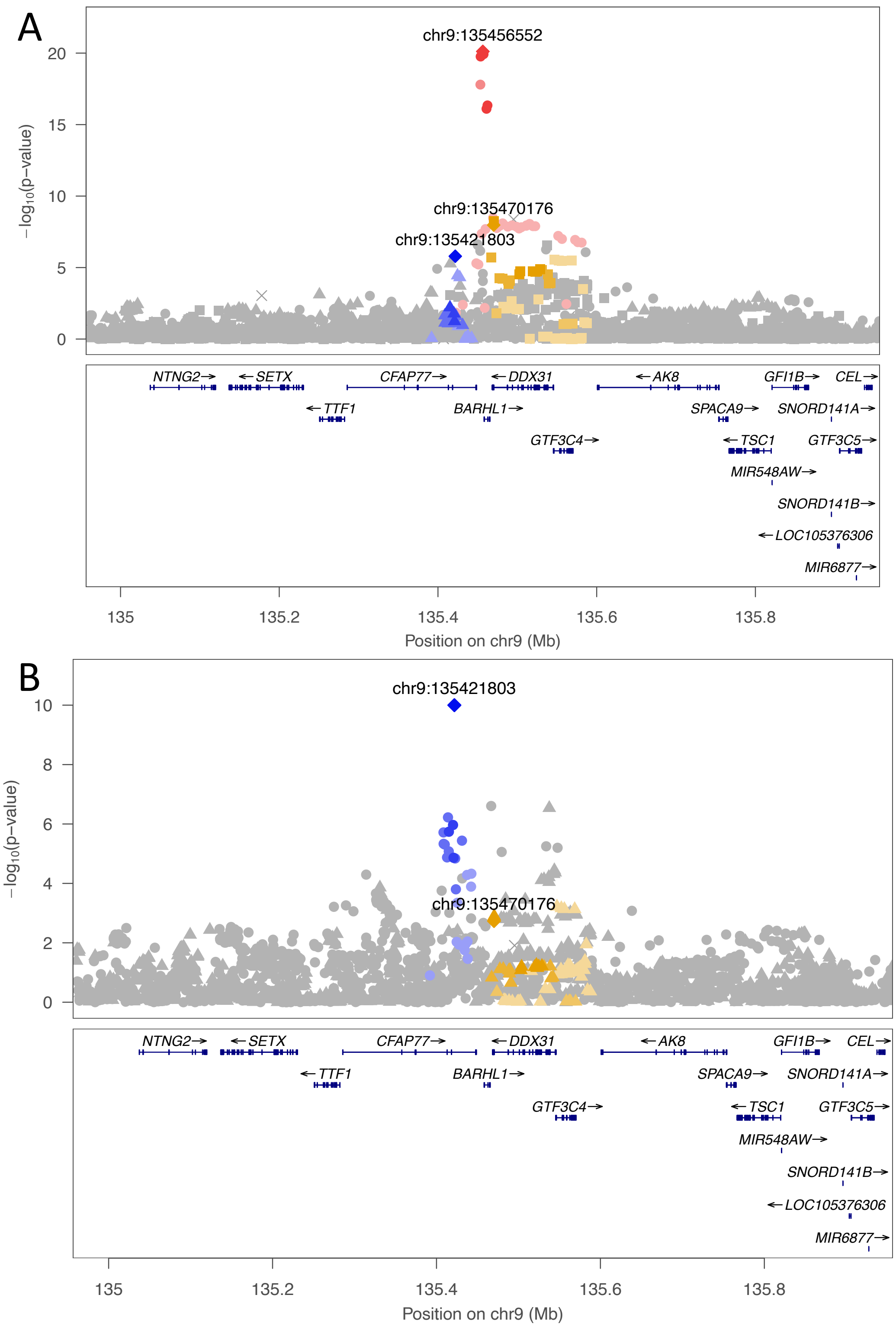

Figure S5: ***DDX31* proinsulin signals.** A: The two conditionally distinct proinsulin signals at the *DDX31* locus, shown in red and blue. In yellow, the female-specific *DDX31* signal identified in Strawbridge (2011), lead variant chr9:135470176, that was not identified in this meta-analysis. B: Proinsulin results after conditioning on the first signal. The secondary *DDX31* proinsulin signal is in blue and the female-specific *DDX31* signal identified by Strawbridge is in yellow. The Strawbridge lead is in low LD (r^2 = 0.09) with the primary *DDX31* lead variant (chr9:135456552), and does not reach the significance threshold after conditioning on the primary signal or conditioning on both the primary and secondary signals.

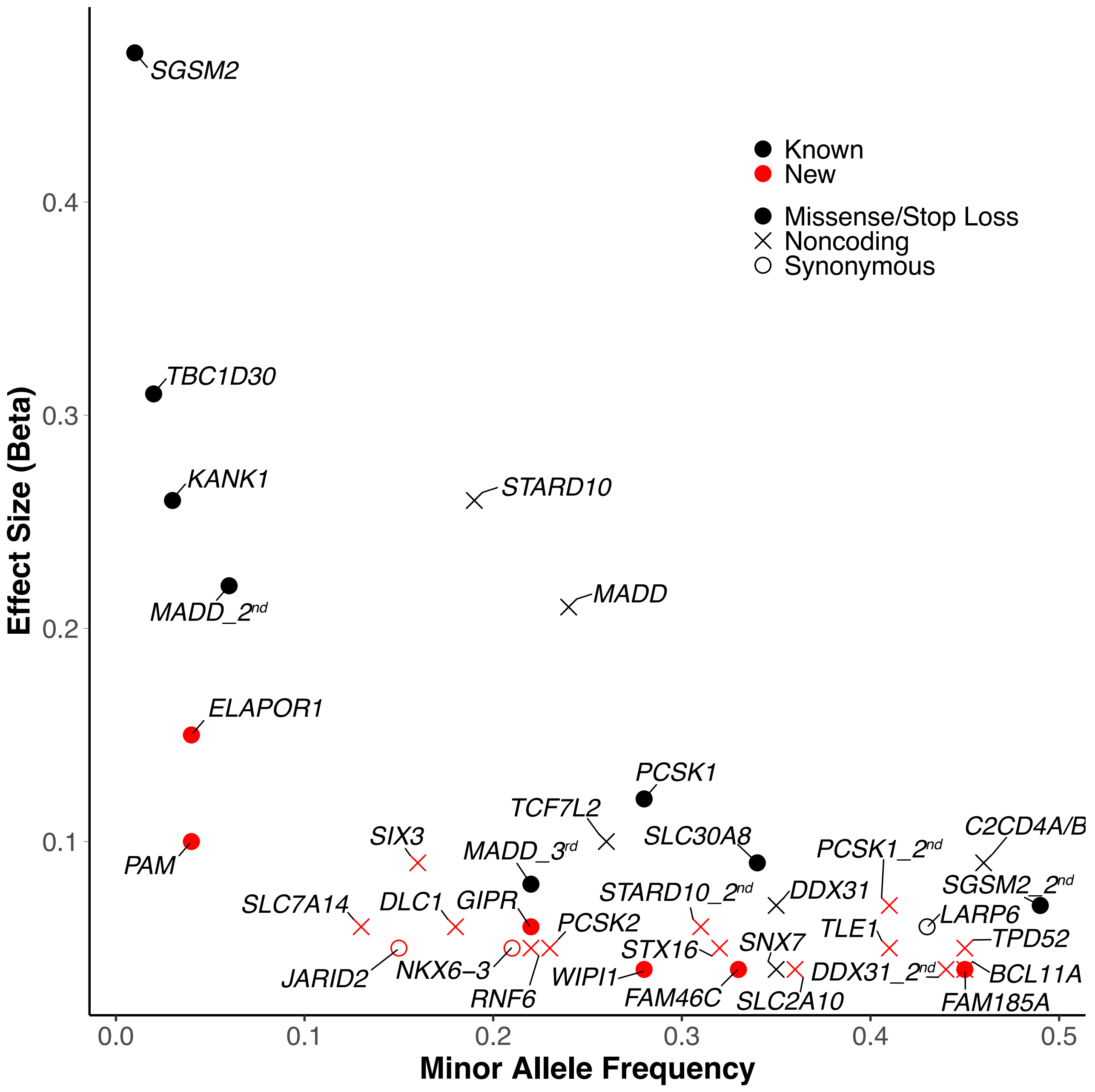

Figure S6a: Effect size versus minor allele frequency of lead proinsulin-associated variants. Dots representing signals that have been identified previously are shown in black and novel proinsulin signals are shown in red. Filled circles indicate loci with a missense or stop loss variant in the extended credible set; open circles indicate loci with a synonymous variant in the extended credible set; x indicates loci with only noncoding variants in the extended credible set.

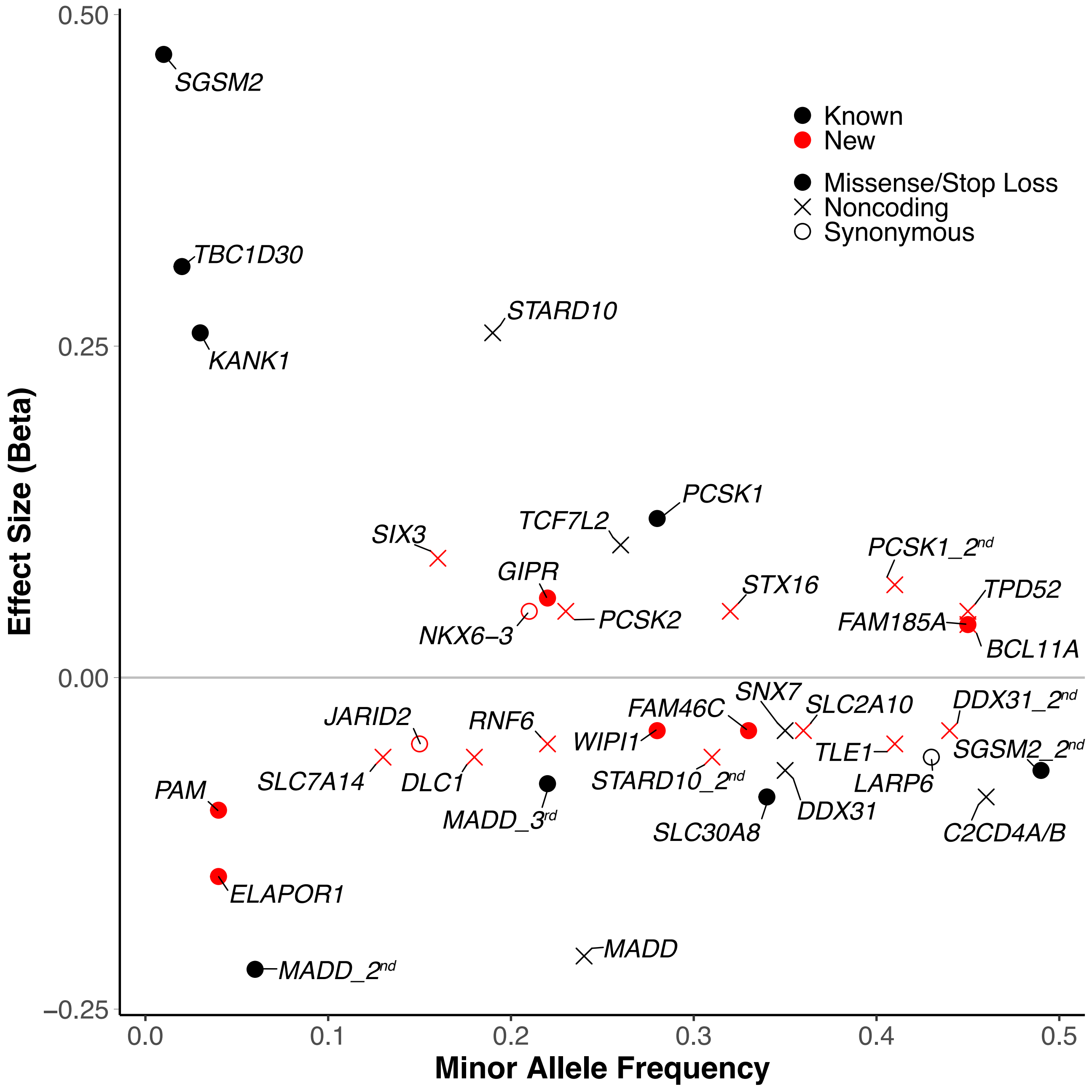

Figure S6b: Effect size versus minor allele frequency of lead proinsulin-associated variants. Effect size is flipped to the minor allele. Dots representing signals that have been identified previously are shown in black and novel proinsulin signals are shown in red. Filled circles indicate loci with a missense or stop loss variant in the extended credible set; open circles indicate loci with a synonymous variant in the extended credible set; x indicates loci with only noncoding variants in the extended credible set.

TBC1D30

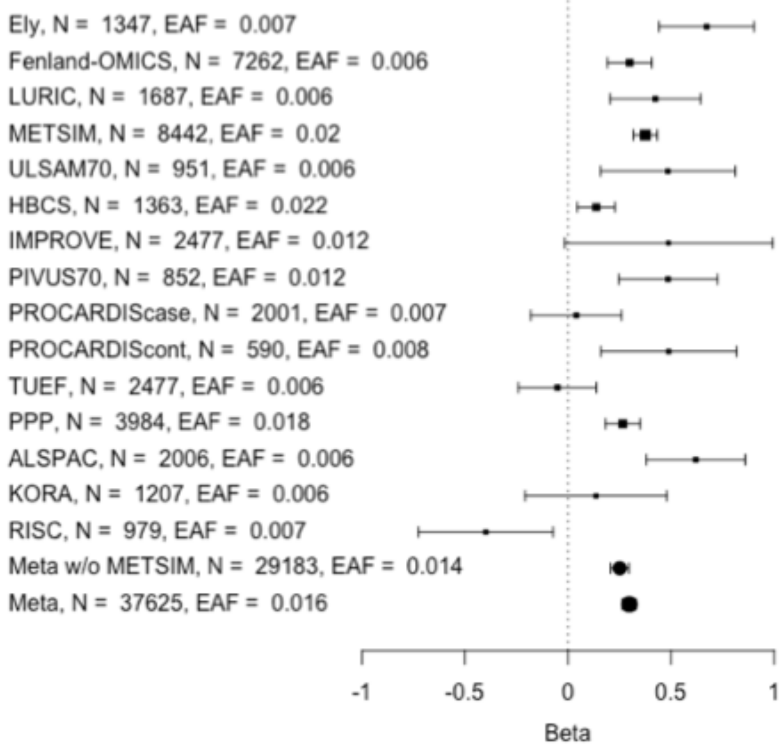

SGSM2

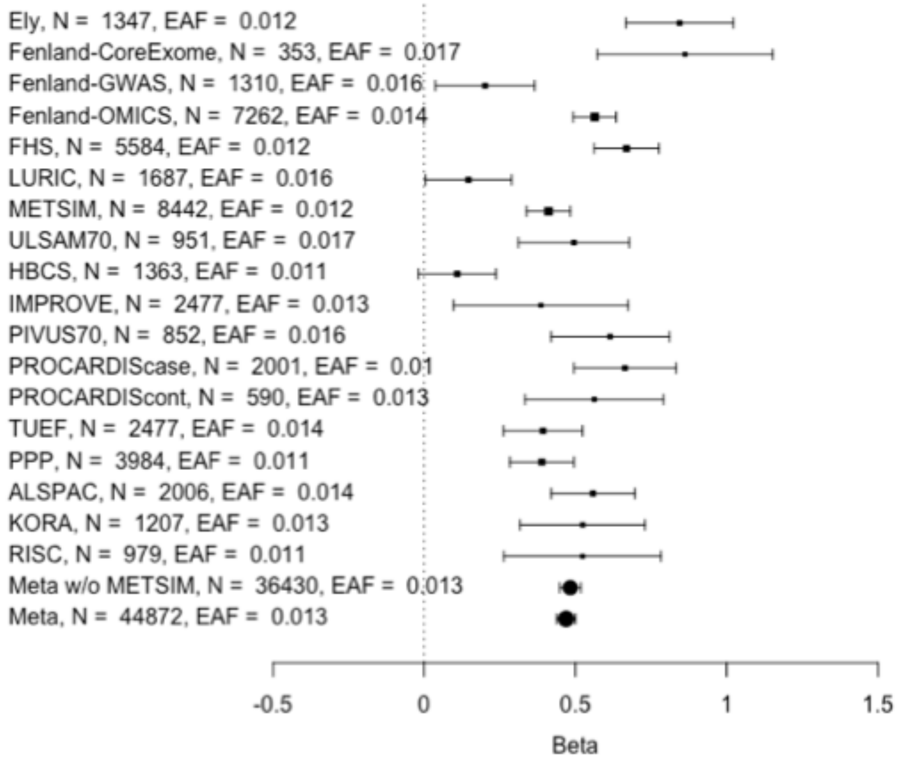

Figure S7: Replication of low frequency proinsulin-associated variants first identified in Huyghe (2013). We replicated the four low-frequency proinsulin-associated variants described in the METSIM exome array study. Continued on next page.

KANK1

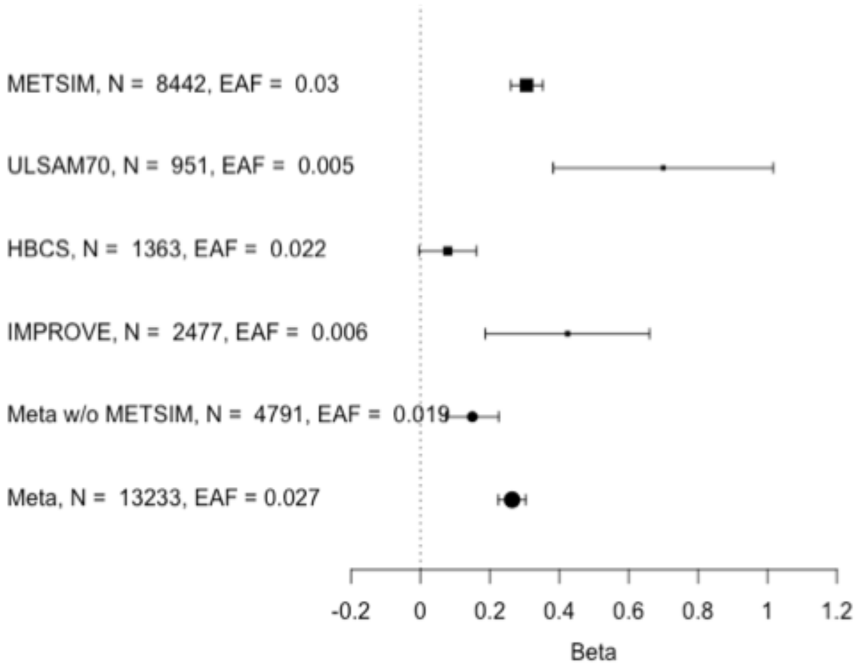

MADD

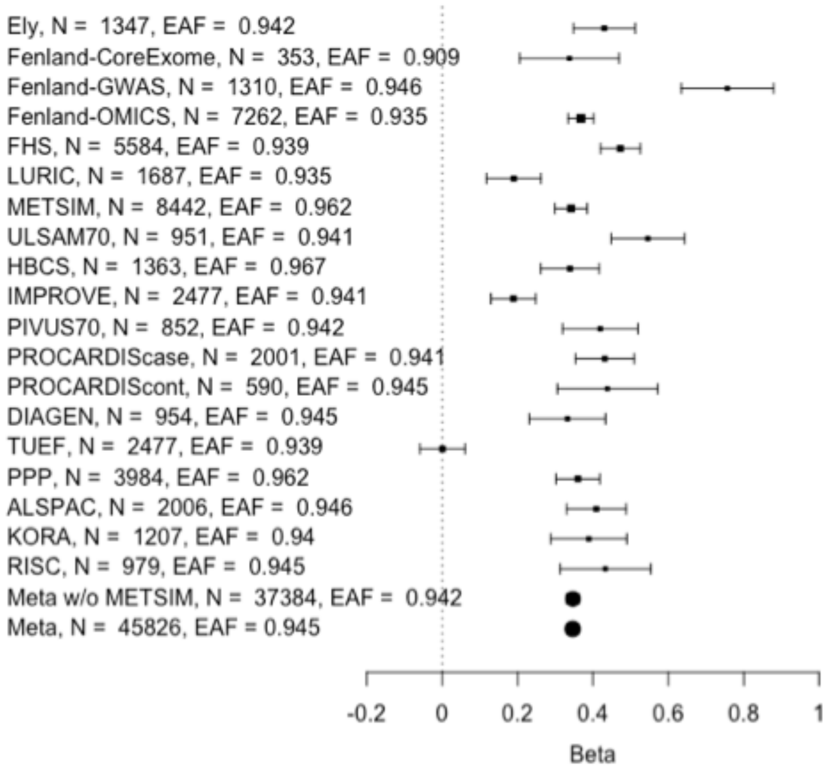

Figure S7 (Continued): **Replication of low frequency proinsulin-associated variants first identified in Hughye (2013).** We replicate the four low-frequency proinsulin-associated variants described in the METSIM exome array study.

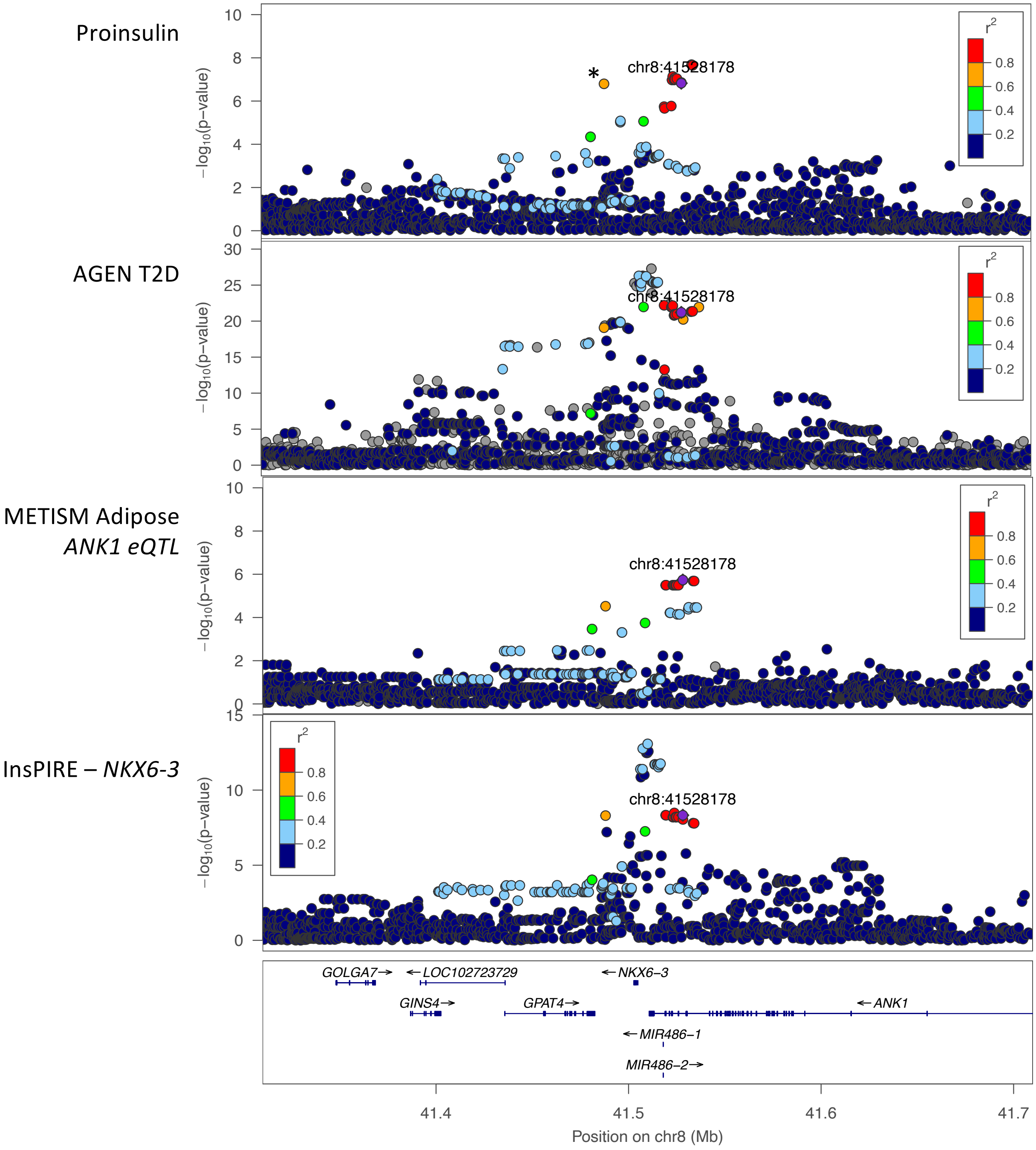

Figure S8: *ANK1/NKX6-3* locus associated with proinsulin and T2D in AGEN EAS, the InsPIRE *NKX6-3* islet eQTL and METSIM *ANK1* adipose eQTL data. Plots shown colored by lead variant for METSIM adipose eQTL. The proinsulin signal colocalizes with the *ANK1* adipose eQTL signal and the secondary AGEN T2D signal. Continued on next page. * chr8:41523745 shown in yellow (beta = 0.042, MAF = 0.23) has a larger sample size than the lead variant chr8:41533514 or LD proxy chr8:41528178 (beta = 0.046, MAF = 0.21) (n = 45,826 vs 44,872). Continued on next page.

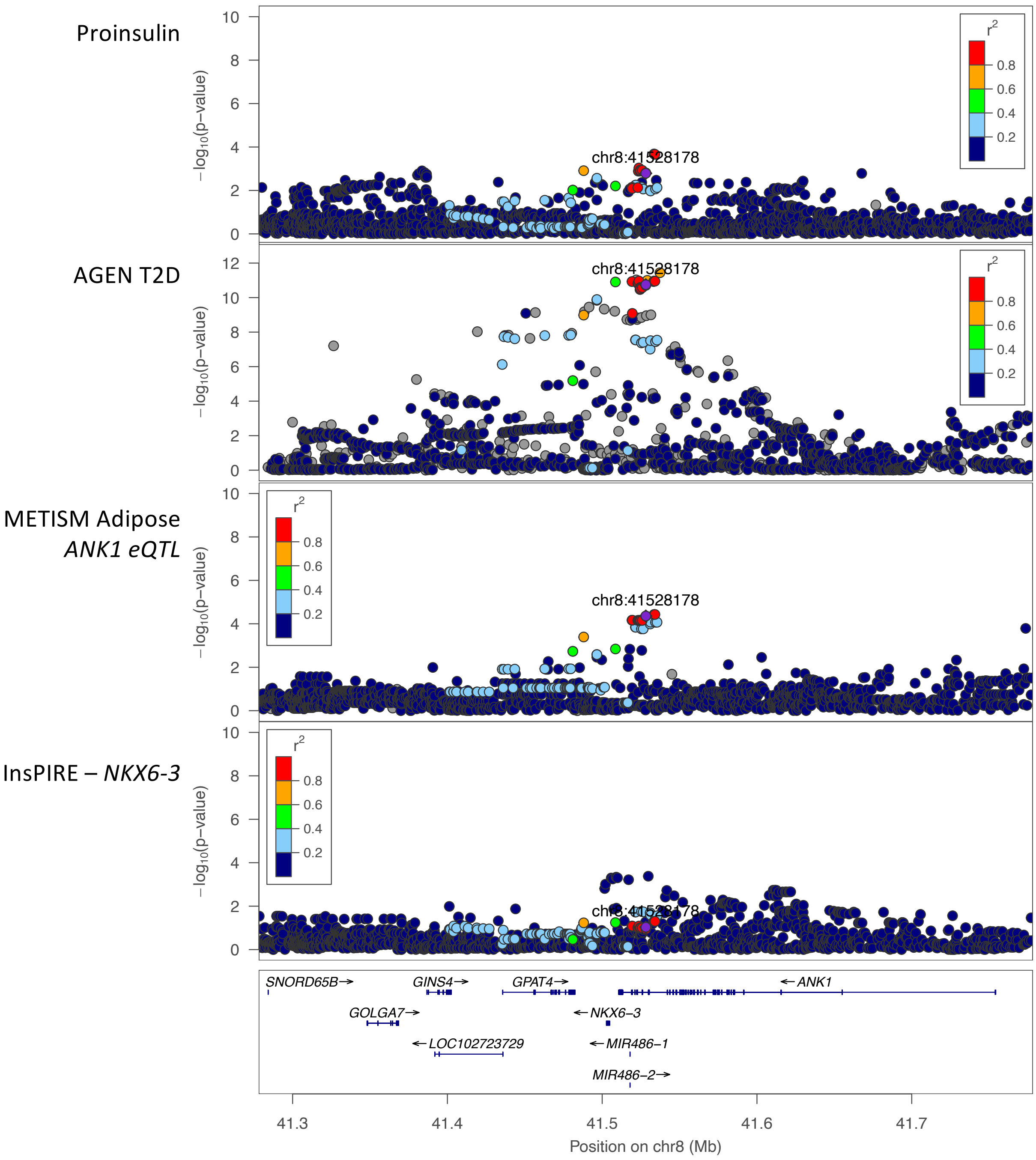

Figure S8 Continued: *ANK1/NKX6-3* locus in proinsulin and T2D AGEN EAS, the InsPIRE *NKX6-3* eQTL and METSIM *ANK1* eQTL data. All results shown have been conditioned on InsPIRE lead variant chr8:41509915, which colocalizes with the first AGEN T2D signal. Plots shown colored by lead variant for METSIM adipose eQTL. The proinsulin signal colocalizes with the *ANK1* adipose eQTL signal and the secondary AGEN T2D signal. Continued on next page.

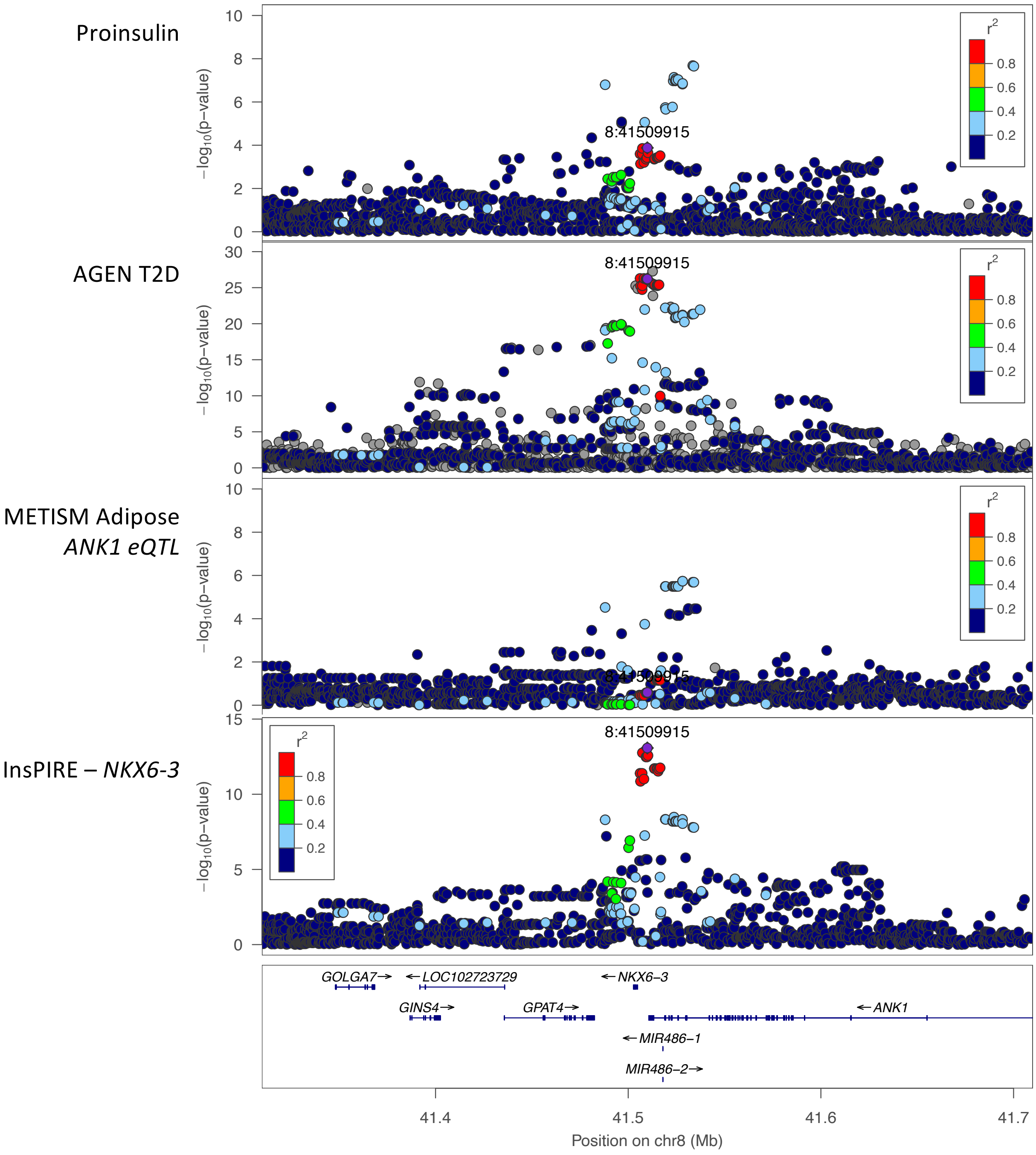

Figure S8 continued: *ANK1/NKX6-3* locus associated with proinsulin and AGEN, the InsPIRE *NKX6-3* eQTL and METSIM *ANK1* eQTL data. Plots shown colored by lead variant for InsPIRE islet eQTL. The proinsulin signal does not colocalize with the InsPIRE *NKX6-3* signal. Continued on next page.

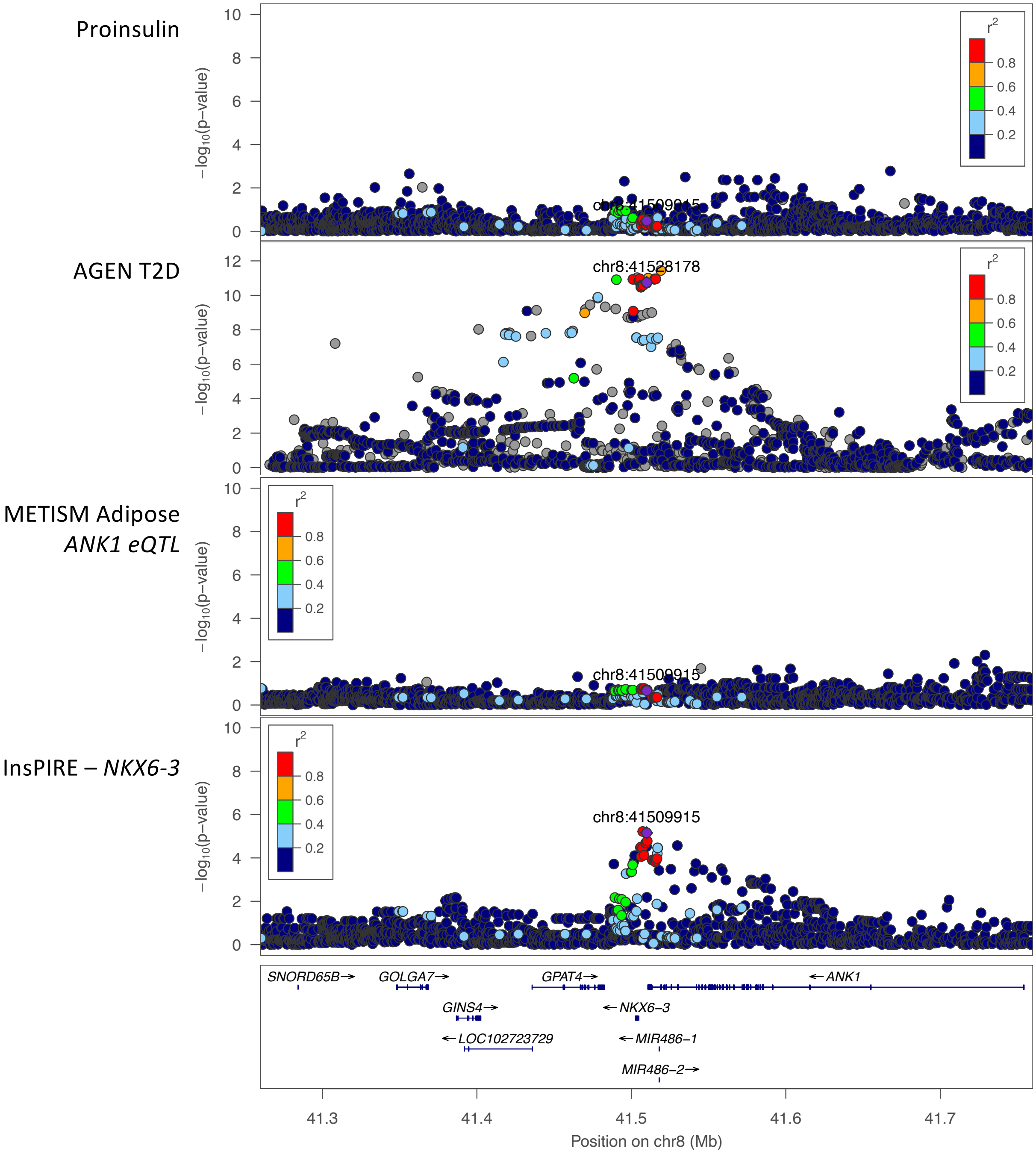

Figure S8 continued: *ANK1/NKX6-3* locus in proinsulin and AGEN, the InsPIRE NKX6-3 eQTL and METSIM *ANK1* eQTL data. All results shown have been conditioned on METSIM lead variant chr8:41533514, which colocalizes with proinsulin and the second AGEN signal. Plots shown colored by lead variant for InsPIRE islet eQTL. The proinsulin signal does not colocalize with the InsPIRE *NKX6-3* signal.

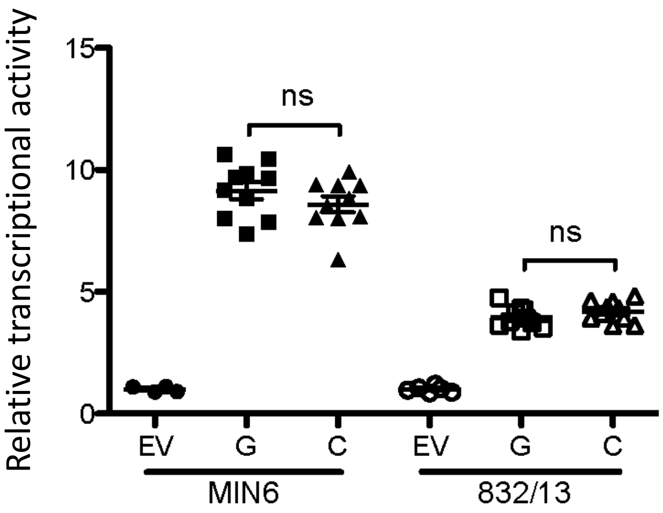

Figure S9: rs10501320 does not exhibit allelic differences in transcriptional activity in the forward orientation. 411-bp fragments including the rs10501320 G or C alleles were cloned upstream of a minimal promoter driving luciferase expression in the forward orientation with respect to the promoter. Values represent fold-change of firefly luciferase/*Renilla* activity normalized to empty pGL4.23 vector in MIN6 and 832/13 cells. EV: empty vector; G/C: alleles at the lead variant rs10501320. In Error bars represent the SEM of four or five independent clones tested across two trials. P-values are calculated from two-sided t-tests.

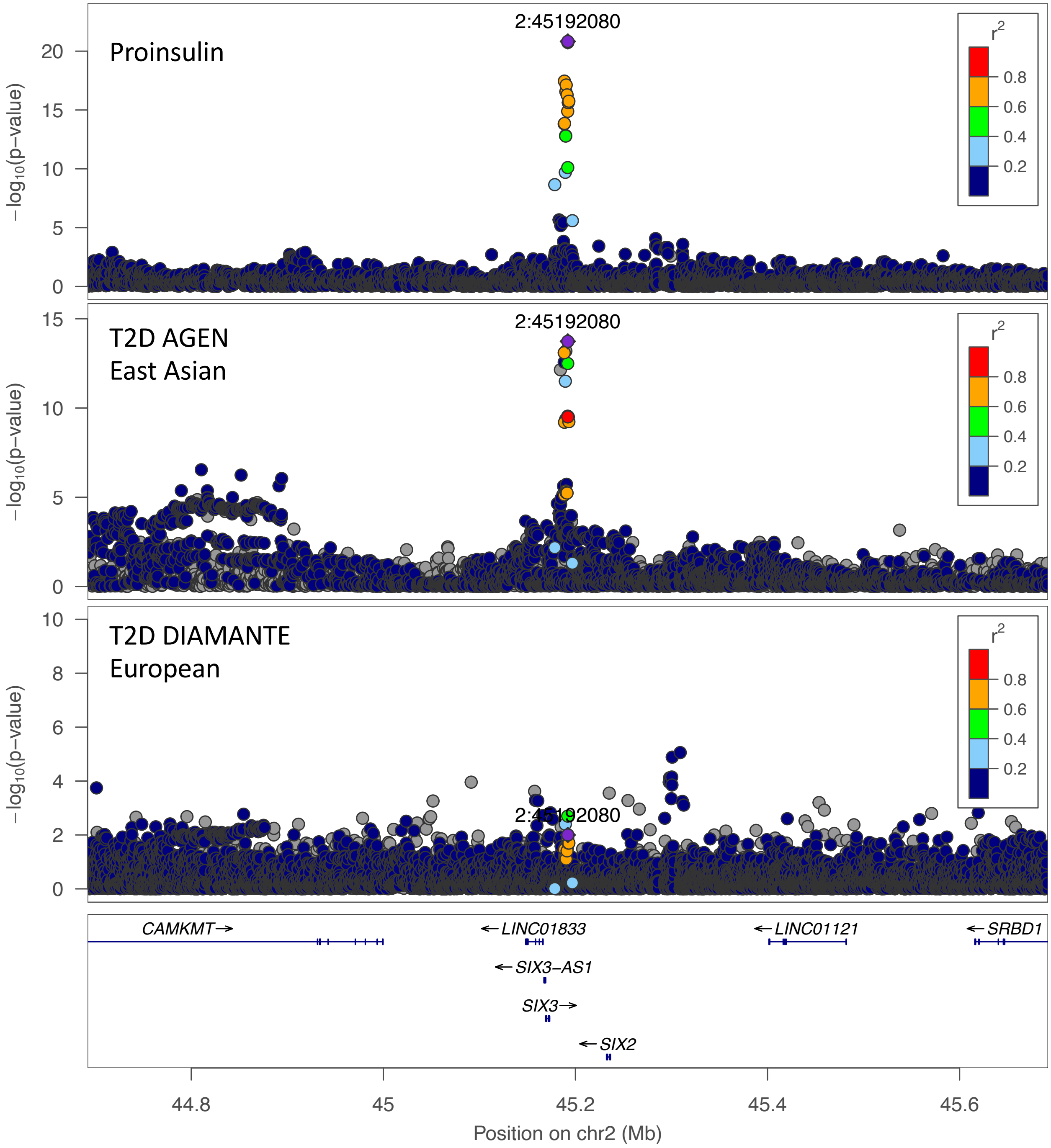

Figure S10: *SIX3* locus associations with proinsulin, T2D DIAMANTE European, and East Asian AGEN T2D. Among East Asians, the *SIX3* locus was associated with T2D; however, despite a common allele frequency in other ancestries, analyses of other ancestries have failed to identify an association between T2D and the *SIX3* locus. Findings in this proinsulin analysis indicates that the glycemic associations at SIX3 are not specific to East Asians and predicts that the direction of effect on glucose levels and T2D in Europeans will be consistent between that shown in East Asians. Continued on next page.

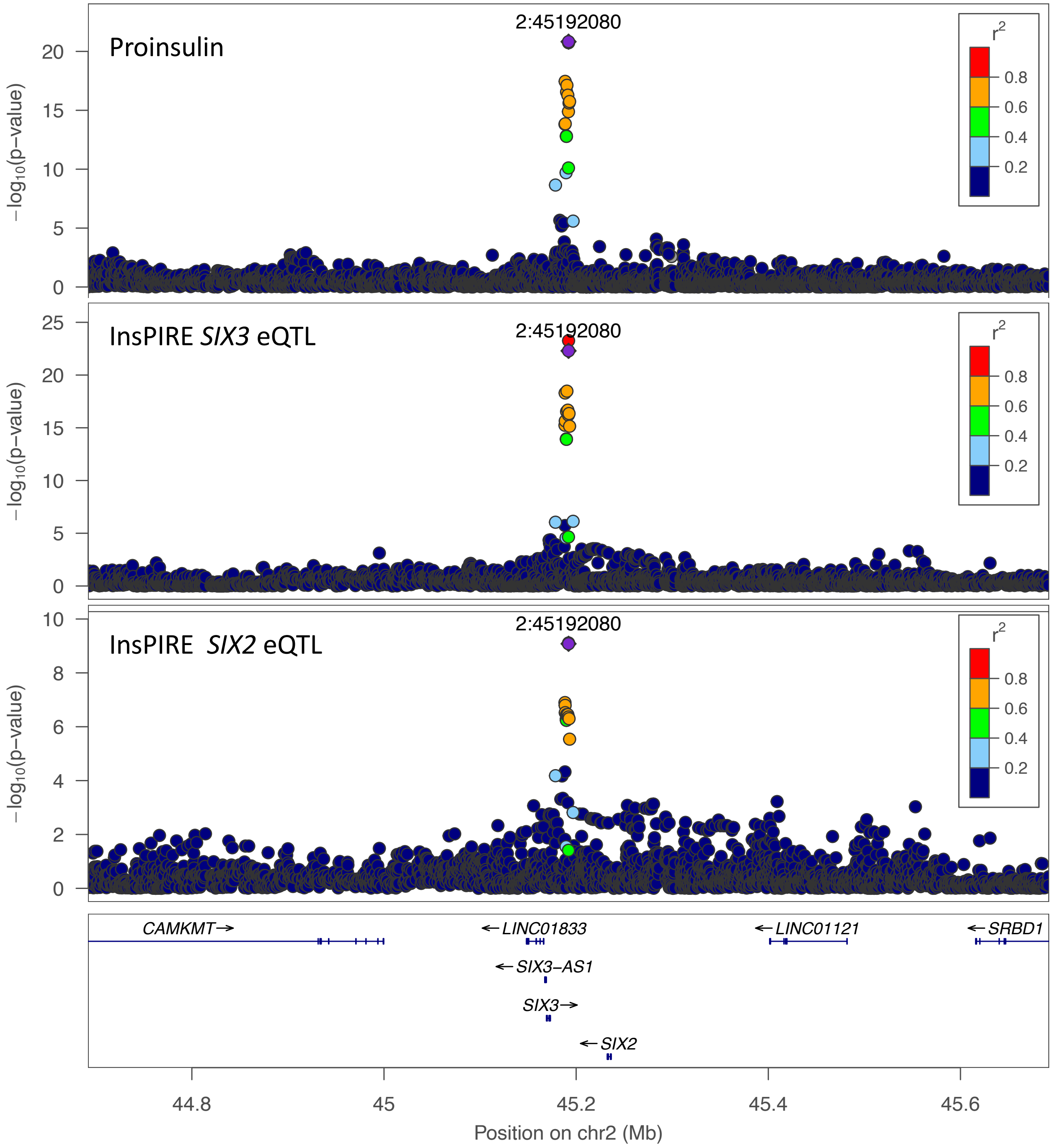

Figure S10 (continued): *SIX3* locus associated with proinsulin and InsPIRE eQTL for *SIX3* and *SIX2* expression levels.

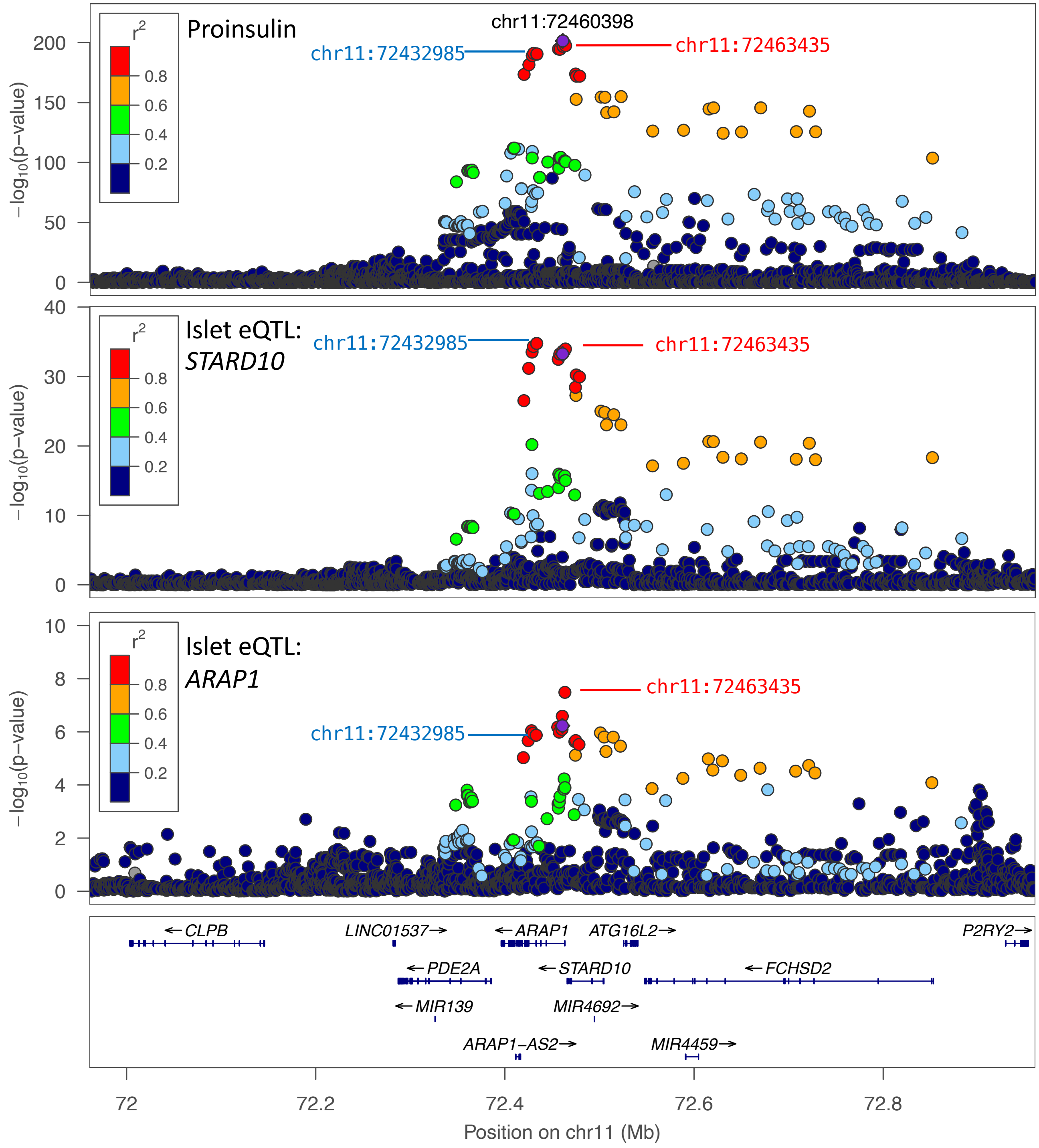

Figure S11: ***STARD10* locus associations with proinsulin and InsPIRE islet *STARD10* and *ARAP1* expression.** Although evidence of colocalization is stronger with *ARAP1* eQTL, the strength of association between the lead variant and *STARD10* expression is stronger. Plots are colored by the lead proinsulin variant (chr11:72460398, rs77464186), denoted by purple diamond. Variant labeled in blue (chr11:72432985) is the lead *STARD10* eQTL variant; variant labeled in red (chr11:72463435) is the lead *ARAP1* eQTL variant.

Cohort Acknowledgements:

ALSPAC: Ethical approval for the study was obtained from the ALSPAC Ethics and Law Committee and the Local Research Ethics Committees. Consent for biological samples has been collected in accordance with the Human Tissue Act (2004). Informed consent for the use of data collected via questionnaires and clinics was obtained from participants following the recommendations of the ALSPAC Ethics and Law Committee at the time. We are extremely grateful to all the families who took part in this study, the midwives for their help in recruiting them, and the whole ALSPAC team, which includes interviewers, computer and laboratory technicians, clerical workers, research scientists, volunteers, managers, receptionists and nurses. The UK Medical Research Council and Wellcome (Grant ref: 217065/Z/19/Z) and the University of Bristol provide core support for ALSPAC. This publication is the work of the authors and Nic Timpson and David Hughes will serve as guarantors for the contents of this paper. This research was funded in whole, or in part, by the Wellcome Trust [202802/Z/16/Z]. For the purpose of Open Access, the author has applied a CC BY public copyright licence to any Author Accepted Manuscript version arising from this submission. **Ely**: The Ely study was supported by the Medical Research Council (MC_UU_12015/1) and NHS Research and Development. **The Fenland Study:** The Fenland Study (10.22025/2017.10.101.00001) is funded by the Medical Research Council (MC UU 12015/1). We are grateful to all the volunteers and to the General Practitioners and practice staff for assistance with recruitment. We thank the Fenland Study Investigators, Fenland Study Co-ordination team and the Epidemiology Field, Data and Laboratory teams. We further acknowledge support for genomics from the Medical Research Council (MC PC 13046). **FHS:** The Framingham Heart Study (FHS) was supported by the National Heart, Lung and Blood Institute's Framingham Heart Study Contract Nos. N01-HC-25195 and HHSN268201500001I) and its contract with Affymetrix, Inc for genotyping services (Contract No. N02-HL-6-4278), and by NIDDK R01DK078616, U01DK078616, UM1DK078616 and R01HL151855. **HBCS:** We thank all study participants as well as everybody involved in the Helsinki Birth Cohort Study. Helsinki Birth Cohort Study has been supported by grants from the Academy of Finland, the Finnish Diabetes Research Society, Folkhälsan Research Foundation, Novo Nordisk Foundation, Finska Läkaresällskapet, Juho Vainio Foundation, Signe and Ane Gyllenberg Foundation, University of Helsinki, Ministry of Education, Jalmari ja Rauha Ahokas foundation, Emil Aaltonen Foundation, and Yrjö Jahnsson foundation. **IMPROVE:** IMPROVE was supported by the European Commission (Contract number: QLG1-CT-2002-00896), the Swedish Heart-Lung Foundation, the Swedish Research Council (projects 8691 and 09533), the Knut and Alice Wallenberg Foundation, the Foundation for Strategic Research, the Stockholm County Council (project 592229), the Strategic Cardiovascular and Diabetes Programmes of Karolinska Institutet and Stockholm County Council, the European Union Framework Programme 7 (FP7/2007-2013) for the Innovative Medicine Initiative under grant agreement n° IMI/115006 (the SUMMIT consortium), the Academy of Finland (Grant #110413), the British Heart Foundation (RG2008/08, RG2008/014) and the Italian Ministry of Health (Ricerca Corrente). **KORA:** The KORA study was initiated and financed by the Helmholtz Zentrum München – German Research Center for Environmental Health, which is funded by the German Federal Ministry of Education and Research (BMBF) and by the State of Bavaria. The German Diabetes Center is funded by the German Federal Ministry of Health (Berlin, Germany) and the Ministry of Culture and Science of the state North Rhine-Westphalia (Düsseldorf, Germany) and receives additional funding from the German

Federal Ministry of Education and Research (BMBF) through the German Center for Diabetes Research (DZD e.V.). **LURIC:** We thank all participants of the LURIC study, as well as the study teams who were either temporarily or permanently involved in patient recruitment as well as sample and data handling. We also thank the laboratory staff at the Ludwigshafen General Hospital and the Universities of Freiburg, Ulm, and Heidelberg in Germany. The genotyping of the LURIC participants was supported by the 7th Framework Programs AtheroRemo (grant agreement number 201668) and RiskyCAD (grant agreement number 305739) of the European Union. LURIC received further support by the H2020 Programs TO_AITION (grant agreement number 848146) and TIMELY (grant agreement number 101017424) of the European Union and the Competence Cluster of Nutrition and Cardiovascular Health (nutriCARD), which is funded by the German Federal Ministry of Education and Research (grant agreement number 01EA1808). **METSIM:** The METSIM study was supported by the Academy of Finland (321428) and Sigrid Juselius Foundation. Additional support for genetic analysis was provided by US NIH grants U01DK062370, 1-ZIA-HG000024, R01DK093757, and R01DK072193. **Botnia PPP:** The Botnia Family Study and the PPP Botnia Study have been financially supported by grants from Folkhälsan Research Foundation, the Sigrid Juselius Foundation, The Academy of Finland (grants no. 263401, 267882, 312063 to LG, 312072 to TT), University of Helsinki, Nordic Center of Excellence in Disease Genetics, EU (EXGENESIS, MOSAIC FP7-600914), Ollqvist Foundation, Swedish Cultural Foundation in Finland, Finnish Diabetes Research Foundation, Foundation for Life and Health in Finland, Signe and Ane Gyllenberg Foundation, Finnish Medical Society, Paavo Nurmi Foundation, State Research Funding via the Helsinki University Hospital, Perklén Foundation, Närpes Health Care Foundation and Ahokas Foundation. The study has also been supported by the Ministry of Education in Finland, Municipal Heath Care Center and Hospital in Jakobstad and Health Care Centers in Vasa, Närpes and Korsholm. The research leading to these results has received funding from the European Research Council under the European Union's Seventh Framework Programme (FP7/2007-2013) / ERC grant agreement n° 269045. The skillful assistance of the Botnia Study Group is gratefully acknowledged. **PROCARDIS:** PROCARDIS was supported by the European Community Sixth Framework Program (LSHM-CT- 2007-037273), AstraZeneca, the Swedish Research Council (8691), the Knut and Alice Wallenberg Foundation, the Swedish Heart-Lung Foundation, the Torsten and Ragnar Söderberg Foundation, the Strategic Cardiovascular Program of Karolinska Institutet and Stockholm County Council, the Foundation for Strategic Research and the Stockholm County Council (560283). IMPROVE was funded by the European Commission (LSHM-CT- 2007-037273), the Swedish Heart-Lung Foundation, the Swedish Research Council (8691), the Knut and Alice Wallenberg Foundation, the Foundation for Strategic Research, the Torsten and Ragnar Söderberg Foundation, the Strategic Cardiovascular Programme of Karolinska Institutet and the Stockholm County Council and the Stockholm County Council (560183). **RISC:** The RISC study was supported by the EU 5th Framework (EU contract QLG1-CT-2001-01252) with additional funding from Astra Zeneca.

Individual acknowledgements:

KAB: US National Institutes of Health (NIH) T32HL129982. A.W. holds a PhD studentship supported by Wellcome Trust. EPW: US NIH T32GM067553. VAP: US NIH T32GM007092. C.M.L is supported by the Li Ka Shing Foundation, NIHR Oxford Biomedical Research Centre, Oxford, NIH (1P50HD104224-01), Gates Foundation (INV-024200), and a Wellcome Trust

Investigator Award (221782/Z/20/Z). CTL: NIH R01DK078616, UM1DK078616, R01HL151855. JL: Medical Research Council MC_UU_00006/1 - Etiology and Mechanisms. RJS: Supported by HDR-UK and University of Glasgow LKAS Fellowships. PW: NIH R01DK078616, R01HL15185. ARW is supported by the Academy of Medical Sciences / the Wellcome Trust / the Government Department of Business, Energy and Industrial Strategy / the British Heart Foundation / Diabetes UK Springboard Award [SBF006\1134]. LJC is supported by NJT's Wellcome Trust Investigator Award (202802/Z/16/Z). TMF is supported by the National Institute for Health and Care Research Exeter Biomedical Research Centre. The views expressed are those of the author(s) and not necessarily those of the NIHR or the Department of Health and Social Care. DAH is supported by NJT's Wellcome Trust Investigator Award (202802/Z/16/Z). JCF: NIH K24 DK110550. AF: Deutsche Forschungsgemeinschaft (DFG) Deutsches Zentrum für Diabetesforschung (DZD). ML: Grant from Academy of Finland. JL: The Finnish Society of Sciences and Letters, Academy of Finland. DAL's contribution to this paper is supported by the UK Medical Research Council (MC_UU_00011/1-6) and British Heart Foundation (CH/F/20/90003 and AA/18/7/34219). JBM: NIH R01DK078616, UM1DK078616, R01HL151855. NJT is a Wellcome Trust Investigator (202802/Z/16/Z), is the PI of the Avon Longitudinal Study of Parents and Children (MRC & WT 217065/Z/19/Z), is supported by the University of Bristol NIHR Biomedical Research Centre (BRC-1215-2001), the MRC Integrative Epidemiology Unit (MC_UU_00011/1) and works within the CRUK Integrative Cancer Epidemiology Programme (C18281/A29019). RW: Deutsche Forschungsgemeinschaft (DFG) Deutsches Zentrum für Diabetesforschung (DZD). NJW: Medical Research Council MC_UU_00006/1 - Etiology and Mechanisms. H.W. acknowledges BHF, Wellcome Trust core award (090532/Z/09/Z, 203141/Z/16/Z, 201543/B/16/Z); HEALTH-F2-2013-601456 (CVGenes@Target), VIAgenomics (SP/19/2/344612) and Oxford BHF Centre of Research Excellence (RE/13/1/30181). SCJP: R01 DK117960. KLM: US NIH UM1 DK126185, R01 DK072193, R01 DK093757.
